# Electrical synapses between mushroom body neurons are critical for consolidated memory retrieval in *Drosophila*

**DOI:** 10.1371/journal.pgen.1008153

**Published:** 2019-05-09

**Authors:** Wei-Huan Shyu, Wang-Pao Lee, Meng-Hsuan Chiang, Ching-Ching Chang, Tsai-Feng Fu, Hsueh-Cheng Chiang, Tony Wu, Chia-Lin Wu

**Affiliations:** 1 Graduate Institute of Biomedical Sciences, College of Medicine, Chang Gung University, Taoyuan, Taiwan; 2 Department of Biochemistry, College of Medicine, Chang Gung University, Taoyuan, Taiwan; 3 Department of Applied Chemistry, National Chi Nan University, Nantou, Taiwan; 4 Department of Pharmacology, College of Medicine, National Cheng Kung University, Tainan, Taiwan; 5 Department of Neurology, Chang Gung Memorial Hospital, Linkou, Taiwan; Katholieke Universiteit Leuven, BELGIUM

## Abstract

Electrical synapses between neurons, also known as gap junctions, are direct cell membrane channels between adjacent neurons. Gap junctions play a role in the synchronization of neuronal network activity; however, their involvement in cognition has not been well characterized. Three-hour olfactory associative memory in *Drosophila* has two components: consolidated anesthesia-resistant memory (ARM) and labile anesthesia-sensitive memory (ASM). Here, we show that knockdown of the gap junction gene *innexin5 (inx5)* in mushroom body (MB) neurons disrupted ARM, while leaving ASM intact. Whole-mount brain immunohistochemistry indicated that INX5 protein was preferentially expressed in the somas, calyxes, and lobes regions of the MB neurons. Adult-stage-specific knockdown of *inx5* in αβ neurons disrupted ARM, suggesting a specific requirement of INX5 in αβ neurons for ARM formation. Hyperpolarization of αβ neurons during memory retrieval by expressing an engineered halorhodopsin (eNpHR) also disrupted ARM. Administration of the gap junction blocker carbenoxolone (CBX) reduced the proportion of odor responsive αβ neurons to the training odor 3 hours after training. Finally, the α-branch-specific 3-hour ARM-specific memory trace was also diminished with CBX treatment and in *inx5* knockdown flies. Altogether, our results suggest INX5 gap junction channels in αβ neurons for ARM retrieval and also provide a more detailed neuronal mechanism for consolidated memory in *Drosophila*.

## Author summary

One of the most important questions in the neuroscience is how the brain process memory. Memory formation requires neuronal communication in the brain via synaptic transmissions, which include chemical and electrical synapses. Unlike the chemical synapses, the biological functions of electrical synapses in memory formation remain poorly understood. Here, we revealed that electrical synapses between mushroom body (MB) αβ neurons in *Drosophila* are critical for consolidated memory retrieval. We also showed that the electrical synapses are important for the branch-specific modification of calcium influx into the αβ neurons during memory retrieval. Our results provide novel insights into the molecular mechanisms and synaptic networks underlying memory retrieval.

## Introduction

Pavlovian olfactory learning in *Drosophila melanogaster*, the fruit fly, is a well-characterized behavioral paradigm in which flies are subjected to a training session of sequential exposures to two distinct odors (conditioned stimulus, CS) with or without electric foot shock (unconditioned stimulus, US)[1]. The assay involves CS-US coincidence detection in the mushroom bodies (MBs), the olfactory learning and memory centers of the fly brain. The MBs are a pair of neuropils composed of around 2,000 major intrinsic neurons, called the Kenyon cells (KCs), in each brain hemisphere[2]. The dendrites of the MB neurons form a calyx, and their axons project anteriorly through the peduncle to give rise to the αβ, α'β', and γ lobes in the middle brain. Three hours after a single training session consists of two genetically distinct forms of memory, anesthesia-sensitive memory (ASM) and anesthesia-resistant memory (ARM), with each accounting for about half of the retention level[3–5].

Synaptic transmission in the brain has two different modalities, chemical and electrical synapses. Neurons mainly use neurotransmitters or neuropeptides to communicate and regulate one another’s functions, which is mediated by chemical synapses. In contrast, electrical synaptic transmission depends on clusters of intercellular channels called gap junctions, which form the pores approximately 1.2 nm in diameter between neurons[6]. These pore structures allow diffusion of small molecules and ions, thus enabling bidirectional electronic signal transmission between neurons. The synchronization of neuronal activity in the hippocampus is mediated by gap junctions in the mammalian brain, which is critical for memory consolidation[7, 8]. In the human and mouse genomes, 21 and 20 gap junction genes have been identified, respectively[9]. The *connexin* and *pannexin* gap junction gene families are found in vertebrates, whereas the *innexin (inx)* gene family is found in invertebrates[10, 11]. *Drosophila melanogaster* has 8 gap junction genes, named *inx1-inx8*. Our previous study showed that two MB modulatory neurons in the *Drosophila* brain, the anterior paired lateral (APL) and dorsal paired medial (DPM) neurons, formed heterotypic gap junction channels via INX6 and INX7, and that disrupting communication through these gap junctions impaired 3-hour ASM[12]. Moreover, a recent study indicated that gap junctions in αβ, α'β', and MB output neurons (MBON-β'2mp) were involved in *Drosophila* visual learning[13].

To determine whether gap junctions in MB neurons are essential for olfactory memory formation, we knocked down each *innexin* gene in MB neurons and found that only the downregulation of *inx5* specifically disrupted 3-hour ARM. Consistent with this result, whole-mount brain immunostaining showed INX5-positive signals in the somas, calyxes, and lobes of the MBs, suggesting the existence of gap junction channels between MB neurons. Knockdown of *inx5* in αβ, but not α'β' or γ, neurons disrupted ARM, indicating that INX5 in αβ neurons was involved in ARM formation. Furthermore, adult-stage-specific knockdown of *inx5* in αβ neurons disrupted ARM, demonstrating that the ARM deficiency was not caused by defects in MB development. We performed a transient inhibition of the action potential in αβ neurons by expressing an engineered halorhodopsin protein (eNpHR)[14], which acts as a light-driven chloride pump, specifically during memory retrieval, but not during acquisition or consolidation. This also led to the disruption of ARM, suggesting that INX5 was involved in ARM retrieval in αβ neurons. We observed a training-induced increase in the proportion of odor-responsive αβ neurons to the training odor (CS+ odor) 3 hours after conditioning, and this phenomenon was disrupted by treatment with the gap junction blocker carbenoxolone (CBX). Finally, we found increased calcium responses to the training odor in the MB α-lobe branch region 3 hours after conditioning, and this increased calcium response was diminished by both gap junction blocker CBX treatment and in *inx5* knockdown flies. These data suggest that INX5 channels coordinate the MB neuronal activity changes to training odor at 3-hour after odor/shock association. Together, our results show that ARM retrieval in *Drosophila* is mediated by gap junction channels composed of INX5 in αβ neurons.

## Results

### MB INX5 is required for ARM

**Fig 1:**
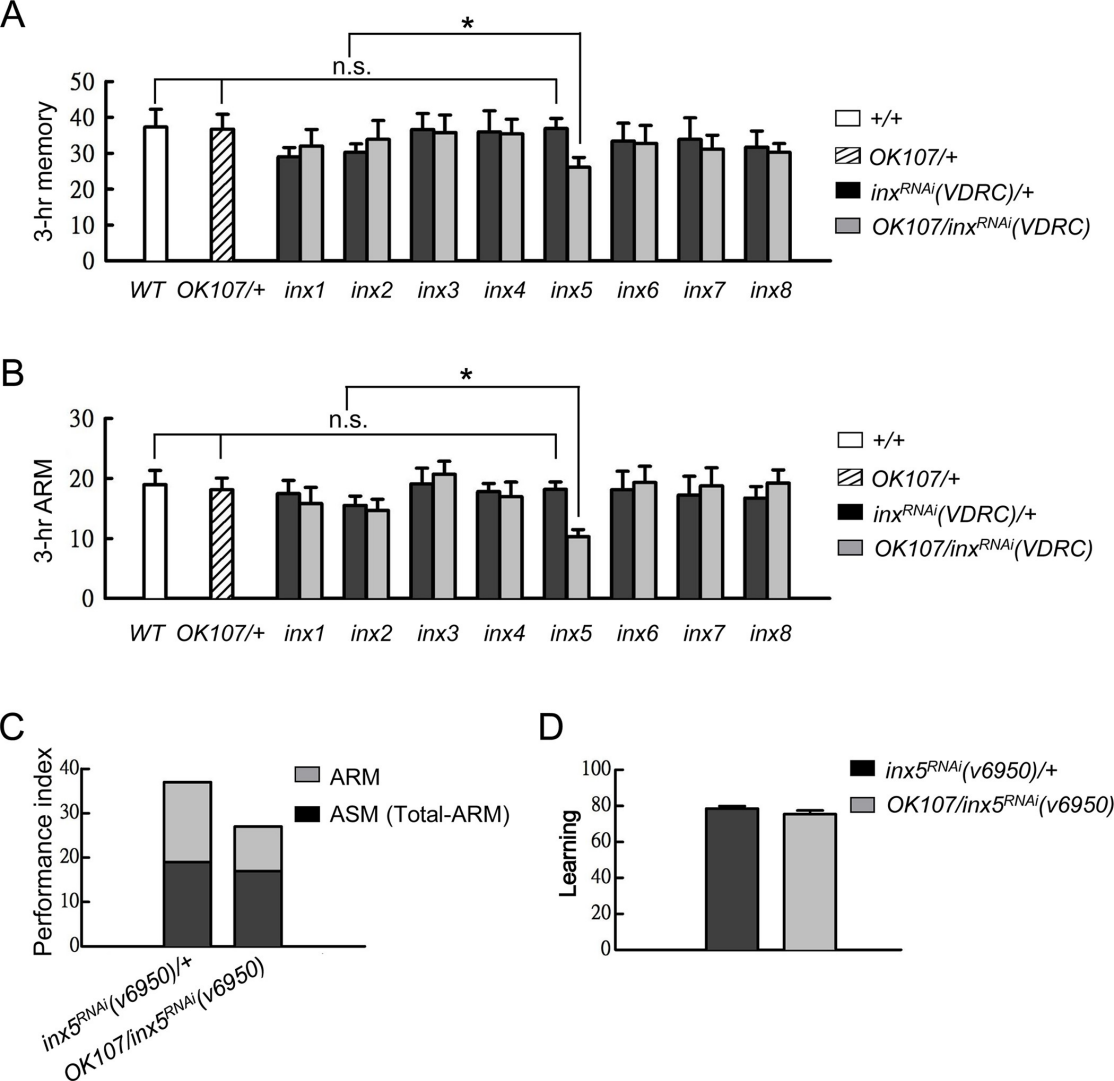
Downregulation of INX5 in MBs impairs ARM. (A) Three-hour memory (performance index) in flies carrying one of the eight *UAS-inx*^*RNAi*^ (Vienna *Drosophila* Resources Center, VDRC) effectors driven by *OK107-GAL4*. Each value represents the mean ± SEM (n = 8). n.s.: not significant (p > 0.05); *, p < 0.05; one-way analysis of variance (ANOVA) followed by Tukey’s test. The VDRC stock numbers for the *UAS-inx*^*RNAi*^ effectors were as follows: *UAS-inx1*^*RNAi*^(v103816), *UAS-inx2*^*RNAi*^ (v102194), *UAS-inx3*^*RNAi*^(v39094), *UAS-inx4*^*RNAi*^(v33277), *UAS-inx5*^*RNAi*^(v6950), *UAS-inx6*^*RNAi*^ (v46398), *UAS*-*inx7*^*RNAi*^(v103256), and *UAS-inx8*^*RNAi*^(v26801). The genotypes were as follows: (1) *+/+*, (2) *OK107-GAL4/+*, (3) +/*UAS-inx*^*RNAi*^(VDRC), and (4) *OK107-GAL4/UAS-inx*^*RNAi*^(VDRC). (B) Three-hour ARM tests were performed on flies carrying the *OK107-GAL4* driver and an *inx* RNAi transgene (VDRC). The flies were trained and tested at 3-hour after training; the 2-min cold shock was applied at 2-hour post-training. Each value represents the mean ± SEM (n = 8–17). n.s.: not significant (p > 0.05); *, p < 0.05; ANOVA followed by Tukey’s test. The genotypes were as follows: (1) *+/+*, (2) *OK107-GAL4/+*, (3) +/*UAS-inx*^*RNAi*^(VDRC), and (4) *OK107-GAL4/UAS-inx*^*RNAi*^(VDRC). (C) The score of ASM, calculated by subtracting ARM from the total 3-hour memory score, was similar to that in the control group, whereas the ARM score (light gray) was reduced in the test group, indicating that ARM was preferentially impaired by INX5 knockdown in MB neurons. The same data as in (A) and (B) are represented. (D) Initial learning was normal in the *inx5*-manipulated flies. Each value represents the mean ± SEM (n = 8; p > 0.05, t-test). The genotypes were as follows: (1) *+/+; UAS-inx5*^*RNAi*^*(v6950)/+*, (2) *+/+; UAS-inx5*^*RNAi*^*(v6950)/+; OK107-GAL4/+*.

In our previous study, we found that heterotypic gap junctions in two MB modulatory neurons, the APL and DPM neurons, are required for 3-hour ASM formation[12]. In addition, the existence of gap junctions in MB neurons was reported in a recent study[13]. We therefore sought to examine whether gap junctions in MB neurons were involved in olfactory memory formation. We used a *Drosophila* RNAi library to express UAS-*inx*^*RNAi*^ transgenes under the control of *OK107-GAL4* to individually silence the *inx* genes in the entire MBs[15, 16]. We found that only *inx5* knockdown disrupted 3-hour memory (Fig 1A and S1A Fig). In the fly, olfactory 3-hour memory consists of the labile ASM and consolidated ARM. We applied 2-min cold-shock anesthetization at 2-hour after training to abolish ASM, and tested the 3-hour ARM retention[5, 17–20]. Interestingly, the memory defect persisted after the cold-shock treatment in *inx5* knockdown flies, suggesting that the memory loss was attributable to the disruption of ARM rather than ASM (Fig 1B–1D; S1B–S1E Fig).

**Fig 2:**
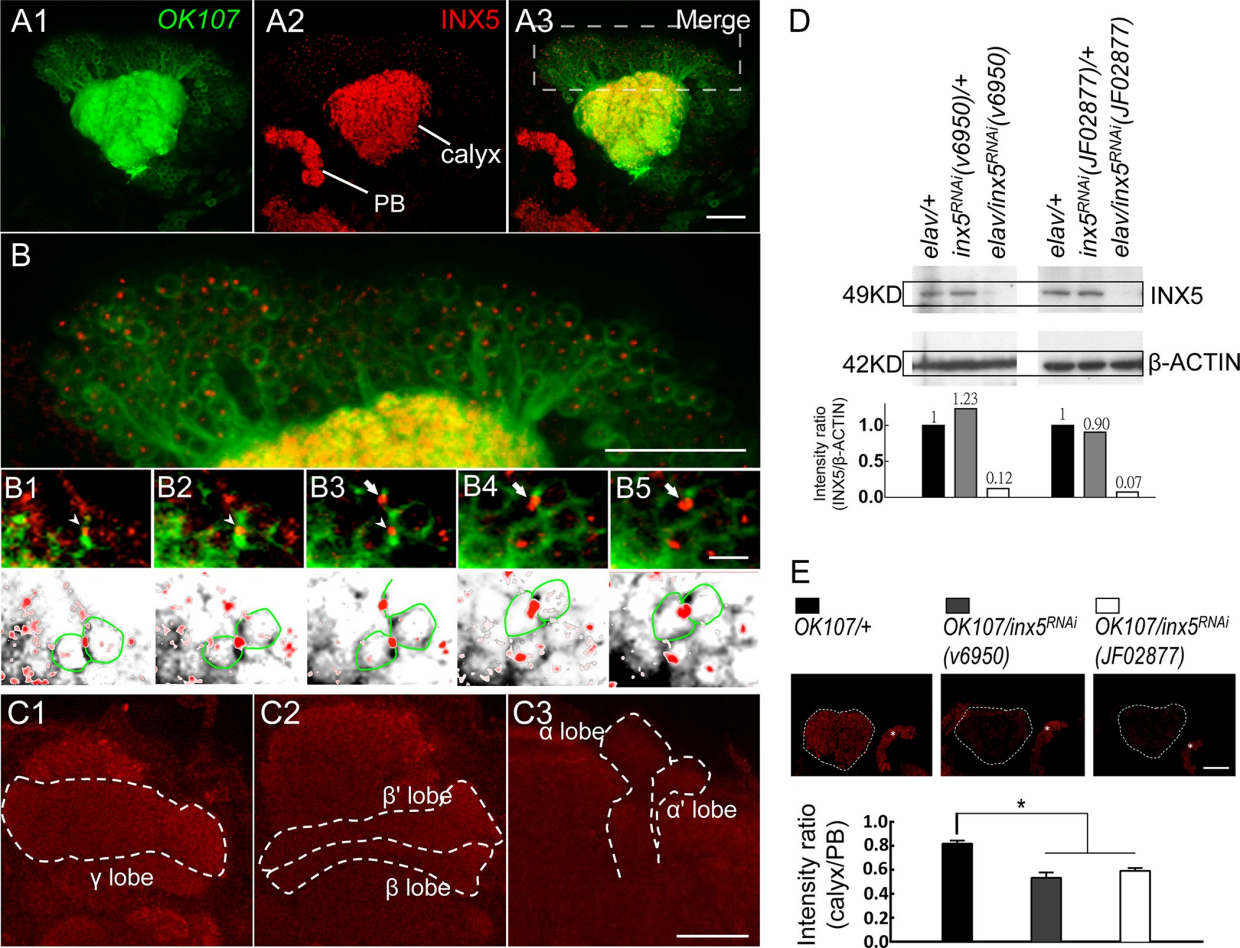
INX5 is expressed in MBs. The *OK107-GAL4*-driven expression of a *UAS-mCD8*::*GFP; UAS-mCD8*::*GFP* reporter (green) in the MBs is shown (A1). Whole-mount adult brain immunostaining of INX5 (red) showed the preferential expression of INX5 in the calyx and protocerebrum bridge (PB) (A2). The merged images show the overlap (yellow) of INX5 staining (red) and GFP reporter (green) in the MB calyx (A3). (B) An enlarged image of the MB neurons (A3), which indicates preferential expression of INX5 in the somas of MB. The scale bar represents 20 μm. (B1-B5) Serial sections show an enlarged confocal image of MB neurons. INX5-positive dots were intercalated in the membrane between MB neurons (arrowheads and arrows) and relatively weak INX5 expression in the nucleus. The genotype in (A-B) was as follows: *+/UAS-mCD8*::*GFP; +/UAS-mCD8*::*GFP; OK107-GAL4/+*. The scale bars represent 10 μm. (C) The INX5 immunostaining signals (red) in distinct lobes of MB neurons. Each lobe showed relatively weak INX5 immunostaining compared to the MB calyx and protocerebrum bridge (also see S2 Fig). The scale bars represent 20 μm. (D) Western blot validation of the specificity of the INX5 antibody and the effectiveness of the *UAS-inx5*^*RNAi*^ effectors driven by pan-neuronal *elav-GAL4*. The genotypes were as follows: (1) *elav-GAL4/+; +/+; +/+*, (2) *+/+; UAS-inx5*^*RNAi*^*(v6950)/+*, (3) *elav-GAL4/+; +/+; UAS-inx5*^*RNAi*^*(v6950)/+*, (4) *+/+; UAS-inx5*^*RNAi*^*(JF02877)/+*, and (5) *elav-GAL4/+; +/+; +/UAS-inx5*^*RNAi*^*(JE02877)*. (E) Quantitative INX5 immunostaining. All images were taken with the same settings. The intensity ratio of INX5 staining represents the difference between the MB calyx and protocerebrum bridge. The scale bars represent 20 μm. Each value represents the mean ± SEM (n = 9–10). *, p < 0.05; one-way analysis of variance followed by Tukey’s test. The genotypes were as follows: (1) *+/+; +/+; OK107-GAL4/+*, (2) *+/+; UAS-inx5*^*RNAi*^*(v6950)/+; OK107-GAL4/+*, and (3) *+/+; +/UAS-inx5*^*RNAi*^*(JF02877); OK107-GAL4/+*.

It has been shown that *inx5* is expressed 50 hours after pupal formation[21]. To further characterize INX5 protein expression in the fly brain, a rabbit polyclonal antibody recognizing *Drosophila* INX5 was generated. Whole-mount brain immunohistochemistry with this antibody showed that INX5 was expressed in the MB calyxes, somas, and lobes (Fig 2A–2C and S2 Fig). Western blotting confirmed that the INX5 protein levels were dramatically decreased in head extracts from two independent *UAS-inx5*^*RNAi*^ flies (*v6950* and *JF02877*) in which the RNA transgene was under the control of the pan-neuronal GAL4 driver, *elav-GAL4* (Fig 2D). Furthermore, quantitative brain immunohistochemistry indicated that two independent *UAS-inx5*^*RNAi*^ flies with the transgene under the control of *OK107-GAL4* had greatly decreased INX5 levels in the MBs (Fig 2E).

### INX5 function in αβ neurons is essential for ARM formation

**Fig 3:**
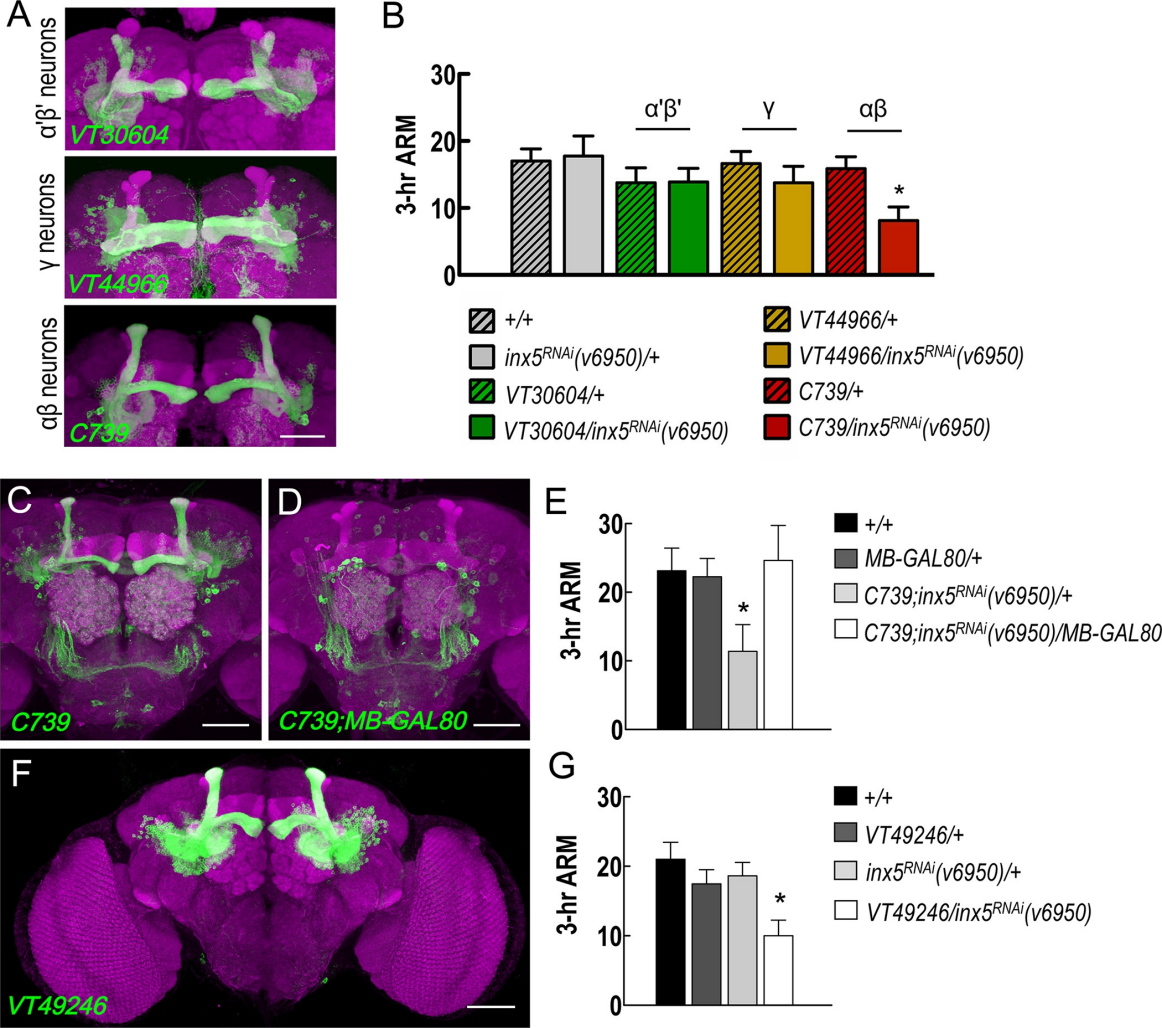
INX5 specifically acts on αβ neurons during ARM formation. (A) Expression patterns (green) of *VT30604-GAL4* (α′β′ neurons), *VT44966-GAL4* (γ neurons), and *C739-GAL4* (αβ neurons). The brain structures were counterstained with a DLG antibody (magenta). The scale bars represent 50 μm. (B) *VT30604-GAL4*, *VT44966-GAL4*, and *C739-GAL4* were used to drive *UAS-inx5*^*RNAi*^ expression in MB α′β′, γ, or αβ neurons, respectively. The 3-hour ARM values for these flies, including controls, are shown. Each value represents the mean ± SEM (n = 8). *, p < 0.05; one-way analysis of variance (ANOVA) followed by Tukey’s test. The genotypes were as follows: (1) *+/+; +/+*, (2) *+/+; UAS-inx5*^*RNAi*^*(v6950)/+*, (3) *+/+; VT30604-GAL4/+*, (4) *+/+; VT30604-GAL4/UAS-inx5*^*RNAi*^*(v6950)*, (5) *+/+; VT44966-GAL4/+*, (6) *+/+; VT44966-GAL4/UAS-inx5*^*RNAi*^*(v6950)*, (7) *C739-GAL4/+; +/+*, and (8) *C739-GAL4/+; +/UAS-inx5*^*RNAi*^*(v6950)*. (C) The expression pattern of *C739-GAL4-*driven GFP expression in the central brain. The brain structures were counterstained with a DLG antibody (magenta). The scale bar represents 50 μm. (D) *C739-GAL4-*driven GFP expression in αβ neurons was reduced using the *MB-GAL80* transgene. The brain structures were counterstained with a DLG antibody (magenta). The scale bar represents 50 μm. (E) ARM defect in the *C739;inx5*^*RNAi*^*(v6950)/+* flies was rescued by removing the expression of GAL4 in αβ neurons using the *MB-GAL80* transgene. Each value represents the mean ± SEM (n = 8) *, p < 0.05; ANOVA followed by Tukey’s test. The genotypes were as follows: (1) *+/+; +/+*, (2) *MB-GAL80/+; +/+*, (3) *C739-GAL4/+; UAS-inx5*^*RNAi*^*(v6950)/+*, and (4) *C739-GAL4/MB-GAL80; +/UAS-inx5*^*RNAi*^*(v6950)*. (F) Expression pattern of *VT49246-GAL4* (αβ neurons). The brain structures were counterstained with a DLG antibody (magenta). The scale bars represent 50 μm. (G) *VT49246-GAL4* was used to drive *UAS-inx5*^*RNAi*^*(v6950)* to confirm the requirement for INX5 in MB αβ neurons for 3-hour ARM formation. Each value represents the mean ± SEM (n = 13–14). *, p < 0.05; ANOVA followed by Tukey’s test. The genotypes were as follows: (1) *+/+; +/+*, (2) *+/+; VT49246-GAL4/+*, (3) *+/+; UAS-inx5*^*RNAi*^*(v6950)/+*, and (4) *+/+; VT49246-GAL4/UAS-inx5*^*RNAi*^*(v6950)*.

**Fig 4:**
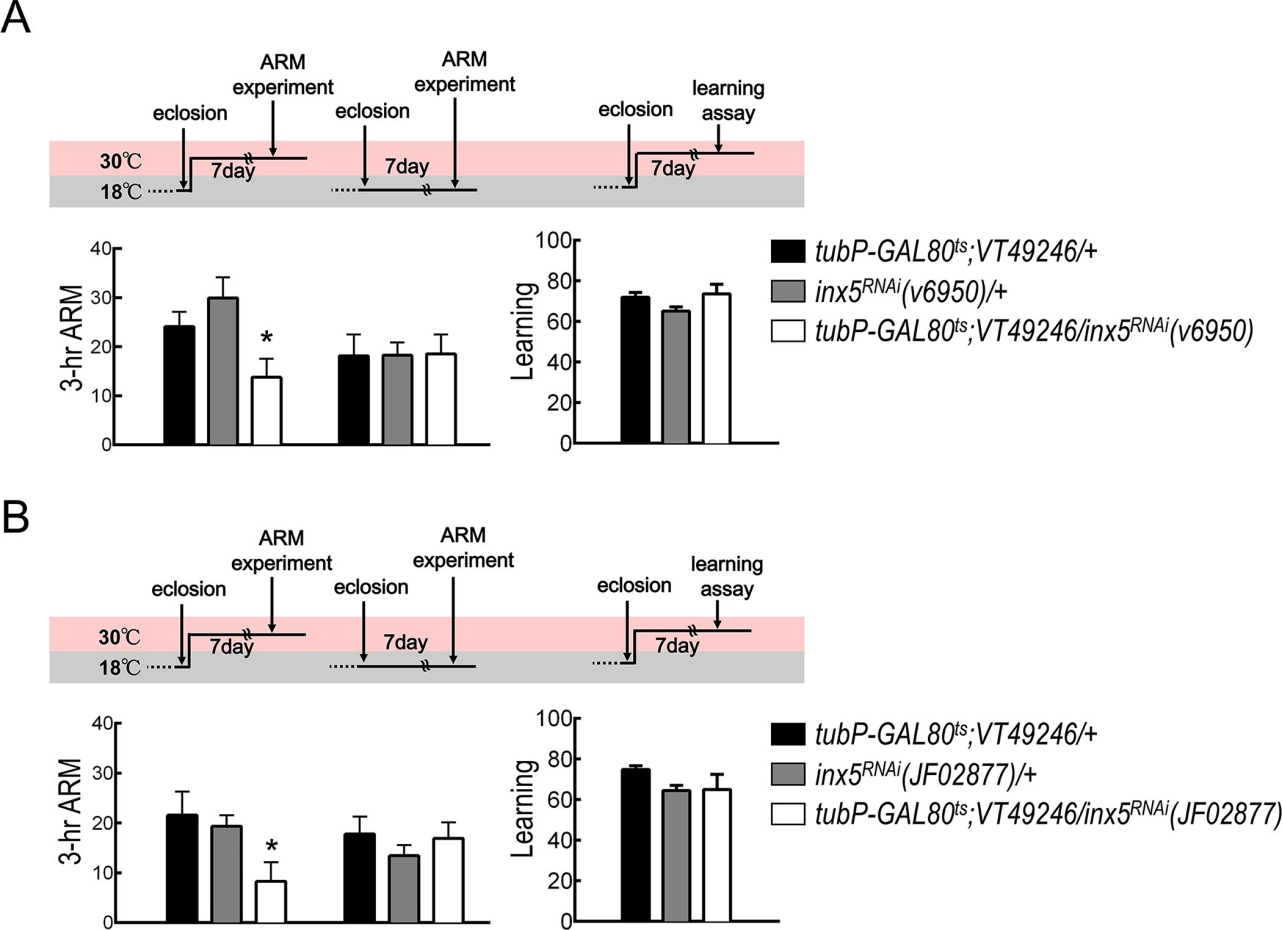
Inducible knockdown of *inx5* in αβ neurons disrupts ARM. (A) Adult-stage-specific INX5 knockdown disrupted ARM but not initial learning. Each value represents the mean ± S.E.M. (n = 8–13). *, p < 0.05; ANOVA followed by Tukey’s test. The genotypes were as follows: (1) *tubP-GAL80*^*ts*^*/+; VT49246-GAL4/+*, (2) *+/+; UAS-inx5*^*RNAi*^*(v6950)/+*, and (3) *tubP-GAL80*^*ts*^*/+; VT49246-GAL4/UAS-inx5*^*RNAi*^*(v6950)*. (B) Additional RNAi effector was used to confirm the role of INX5 in 3-hour ARM formation. Each value represents the mean ± S.E.M. (n = 8–10). *, p < 0.05; ANOVA followed by Tukey’s test. The genotypes were as follows: (1) *tubP-GAL80*^*ts*^*/+; VT49246-GAL4/+*, (2) *+/+; +/UAS-inx5*^*RNAi*^*(JF02877)*, and (3) *tubP-GAL80*^*ts*^*/+; VT49246-GAL4/UAS-inx5*^*RNAi*^*(JF02877)*.

Brain immunostaining showed INX5-positive signals in most if not all MB calyxes and somas. To identify the subset of MB neurons in which INX5 expression is required for ARM formation, we performed behavioral screening using RNAi-mediated *inx5* knockdown with GAL4 drivers specific for different MB neurons. *VT30604-GAL4*, *VT44966-GAL4*, and *C739-GAL4* drive expression of *UAS-inx5*^*RNAi*^ in α′β′, γ, and αβ neurons respectively (Fig 3A). Genetic knockdown of INX5 in αβ neurons, but not in α′β′ or γ neurons, disrupted ARM, suggesting that INX5 in αβ neurons regulates the ARM process (Fig 3B). It is important to consider that *C739-GAL4* is not exclusively expressed in αβ neurons (Fig 3C). We therefore combined the *MB-GAL80* transgene to reduce *GAL4* expression in the MB neurons. The presence of the *MB-GAL80* transgene specifically abolished *GAL4* activity in MB neurons, but left its expression unchanged in non-MB neurons (Fig 3D). Three-hour ARM of *C739-GAL4/MB-GAL80; UAS-inx5*^*RNAi*^*/+* flies were statistically indistinguishable from both wild-type and *MB-GAL80/+* flies and were also statistically different from that of *C739-GAL4/+; UAS-inx5*^*RNAi*^*/+* flies (Fig 3E). Moreover, this behavioral result was further confirmed in an additional GAL4 line (*VT49246-GAL4*) with specifically labeled αβ neurons in the fly brain (Fig 3F and 3G). Furthermore, our previous study found that glutamate release from MB αβ output neurons (MBON-β2β′2a) was required for ARM[20]. To test the possibility that gap junctions between αβ neurons and MBON-β2β′2a are involved in ARM formation, we genetically knocked down each *inx* gene in MBON-β2β′2a and found that all the modified flies displayed normal ARM (S3 Fig). In addition, using a dye-coupling approach, Liu and colleagues found no gap junction connectivity between αβ neurons and their target MBONs[13]. Furthermore, our pervious study found that knockdown of each *inx* gene in the projection neurons and APL neurons via expressing individual *UAS-inx*^*RNAi*^ by *GH146-GAL4* did not impair ARM[12]. Based on these results taken together, we conclude that ARM formation requires gap junctions between αβ neurons. In order to rule out the possibility that the chronic RNA-mediated knockdown of *inx5* causes developmental defects in the MBs, we examined the gross morphologies of the MBs in the *inx5-*manipulated flies and found no significant differences as compared to control flies, suggesting that the MB structure was unaffected by the chronic *inx5* knockdown (S4 Fig). Furthermore, we used an inducible knockdown strategy to silence INX5 expression specifically in the adult stage utilizing a temperature-sensitive GAL80 repressor (*tubP-GAL80*^*ts*^). Inducible knockdown of INX5 still induced significant impairment of 3-hour ARM, but not initial learning (Fig 4), suggesting that INX5 in αβ neurons is required post-developmentally for ARM formation.

### Hyperpolarization of αβ neurons during memory retrieval disrupts ARM

**Fig 5:**
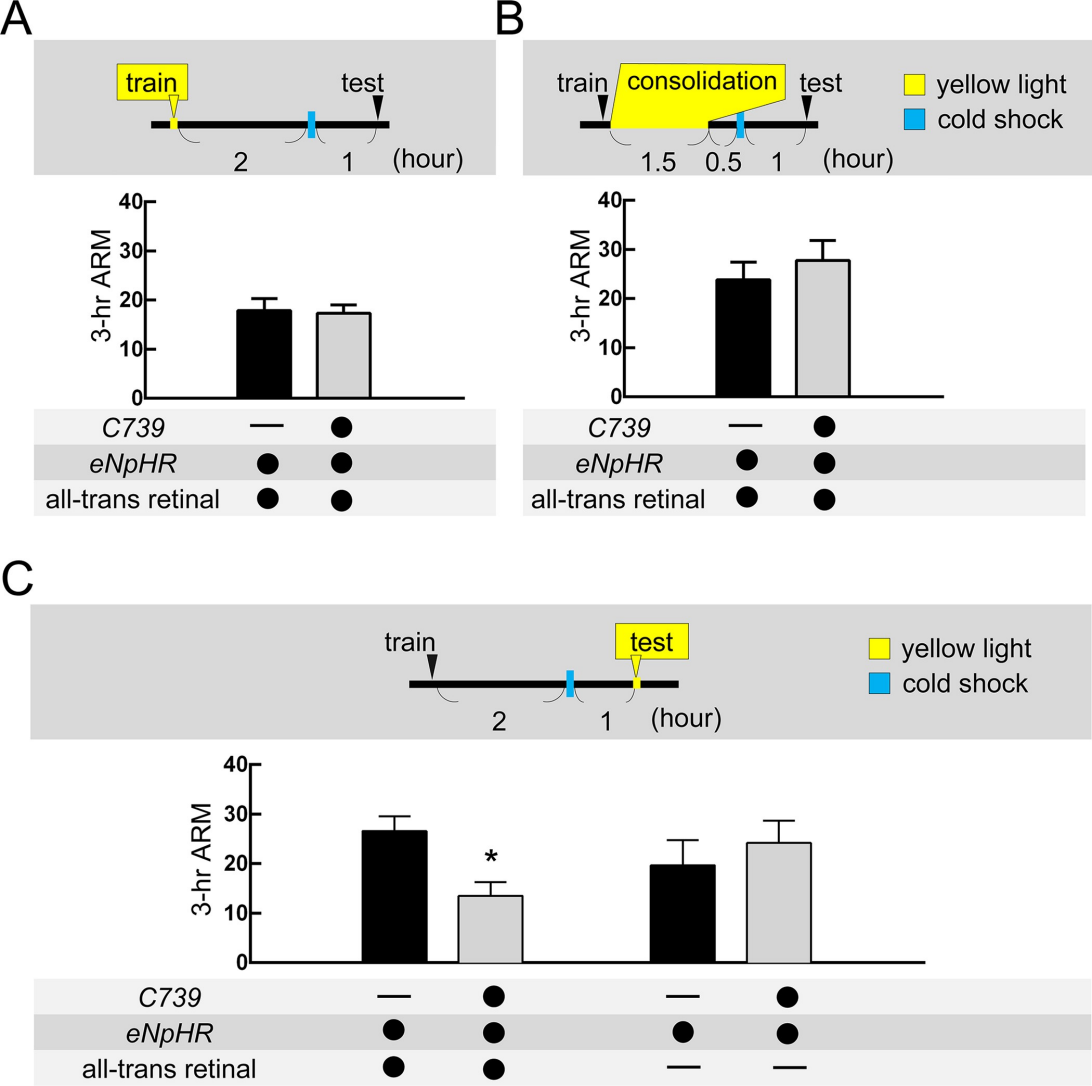
Hyperpolarization of αβ neurons during retrieval but not during acquisition or consolidation, impairs ARM. (A) *eNpHR*-mediated hyperpolarization of αβ neurons during memory acquisition (train) did not affect 3-hour ARM. Each value represents the mean ± SEM (n = 6; p > 0.05, t-test). (B) *eNpHR*-mediated hyperpolarization of αβ neurons during memory consolidation did not affect 3-hour ARM. Each value represents the mean ± SEM (n = 6–9; p > 0.05, t-test). (C) *eNpHR*-mediated hyperpolarization of αβ neurons during memory retrieval (test) impaired 3-hour ARM. Each value represents the mean ± SEM (n = 6–10). *, p < 0.05; t-test. The genotypes were as follows: (1) *+/UAS-eNpHR-YFP; +/UAS-eNpHR-YFP*, (2) *C739-GAL4/UAS-eNpHR-YFP; +/UAS-eNpHR-YFP*.

Consistent with previous studies, blocking chemical synaptic transmission by a temperature sensitive *shibire* (*shi*^*ts*^) in αβ neurons during memory retrieval[20, 22] but not during acquisition or consolidation (S5A Fig), disrupted ARM. The *shibire* gene encodes the *Drosophila* homologue of dynamin, which is required for the fission of endocytic vesicles from the presynaptic membrane. A dominant-negative mutant form of *shibire* interferes with the recycling of neurotransmitters but cannot block the gap junction-mediated propagation of action potential between adjacent neurons[23–27]. We therefore applied optogenetic tools to transiently block action potential in αβ neurons during memory acquisition, consolidation, or retrieval which subsequently allowed us to examine the specific memory phase of ARM that gap junctions were involved in. We overexpressed *eNpHR* to transiently hyperpolarize αβ neurons during different phases of the ARM process. The *eNpHR*-mediated hyperpolarization of αβ neurons during memory retrieval impaired ARM in *C739-GAL4* > *UAS-eNpHR* and *VT49246-GAL4 > UAS-eNpHR* flies. In contrast, *eNpHR*-mediated hyperpolarization of αβ neurons during memory acquisition or consolidation did not impair ARM, suggesting that the activity in αβ neurons is only required for ARM retrieval (Fig 5 and S5B Fig). Although the *eNpHR*-mediated hyperpolarization in αβ neurons also affects the secretion of neuropeptides, blocking these secretions (e.g. *amnesiac*) in αβ neurons does not affect ARM [28]. Knockdown of *inx5* gap junction gene in αβ neurons disrupted ARM and highlights the role of gap junctions between αβ neurons[23–27] (Figs 2, 3, and 4). ARM deficiency was only observed in all-trans-retinal-fed flies indicates that the ARM defect was caused by the transient blockade of αβ neuronal activity through *eNpHR*-mediated neuronal silencing (Fig 5C and S5B Fig). Taken together, these data suggest that INX5 gap junctions are involved in ARM retrieval from αβ neurons in the fly brain.

### INX5 is essential for 3-hour ARM-specific memory trace in αβ neurons

**Fig 6:**
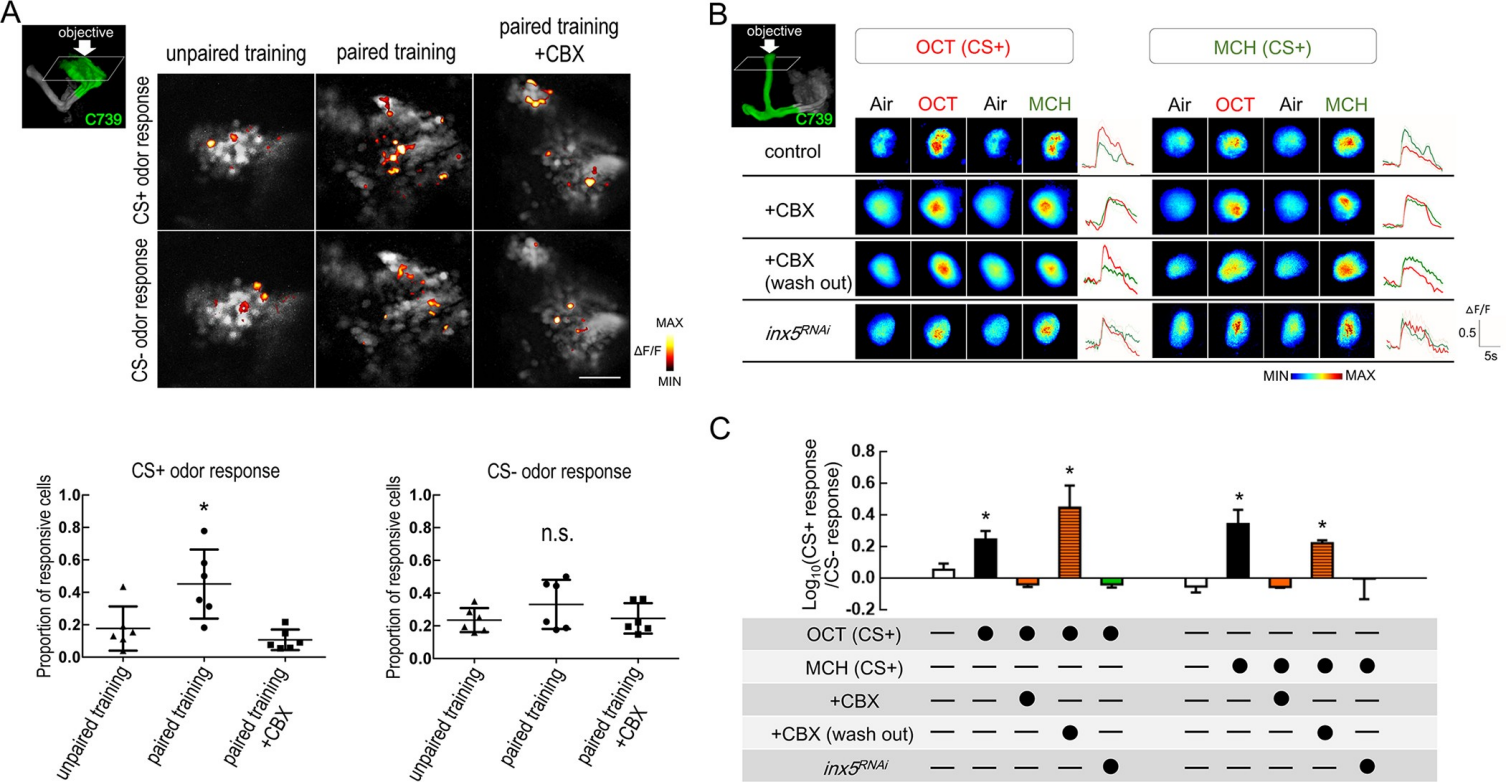
Knockdown of INX5 in αβ neurons disrupts 3-hour ARM-specific memory trace. (A) Top diagram illustrating the paired and unpaired training protocols. The GCaMP6 response at 3-hour after training was assayed after exposure to a 2-min cold shock given at 2-hour after training by placing a plastic vial containing the trained flies in ice water. For the paired training group: flies received CS+ odor with electrical shocks, followed by exposure to the CS- odor without electrical shock. CS- odor was applied after a 1-min exposure to fresh air. For the unpaired training group: flies received CS+ odor without electrical shock, followed by exposure to the CS- odor without electrical shock, and the electrical shocks were applied 1-min later after CS- odor. Odor/shock paired training induced an increase in the proportion of odor responsive αβ neurons to the conditioned (CS+) odor, but not the unconditioned (CS-) odor 3 hours after training, compared to the unpaired training group. The proportion of odor responsive αβ neurons to the CS+ odor was reduced after treatment with the gap junction blocker CBX (MCH as CS+ odor; OCT as CS- odor). *, p < 0.05; one-way analysis of variance followed by Tukey’s test. Genotype: *C739-GAL4/UAS-GCaMP6m; +/+*. (B) Odor/shock paired training (control group) induced an increase in the GCaMP6 responses in the α-lobe axonal branch of the MB neurons to the training odor [OCT-trained flies: OCT (CS+), MCH-trained flies: MCH (CS+)], and this increase was abolished with CBX treatment (row 2, +CBX) or *C739-GAL4 > UAS-inx5*^*RNAi*^*(v6950)* flies (row 4, *inx5*^*RNAi*^ group). The GCaMP6 responses were recorded 3 hours after training with a 2-min cold shock given at 2-hour postconditioning. (C) Quantification of the enhanced GCaMP6 responses to the training odor (CS+) relative to the non-training odor (CS-) in the α-lobe in OCT-trained (left panel) or MCH-trained (right panel) flies. The recordings were performed in the α-lobe tips. The Log ratios of the CS+ response to the CS- response were calculated using the peak response amplitudes. Each value represents the mean ± SEM (n = 8–10). *, p < 0.05; one-way analysis of variance followed by Tukey’s test. Genotypes: (1) *C739-GAL4/UAS-GCaMP6m; +/+*, (2) *C739-GAL4/UAS-GCaMP6m; UAS-inx5*^*RNAi*^*(v6950)/+*.

**Fig 7:**
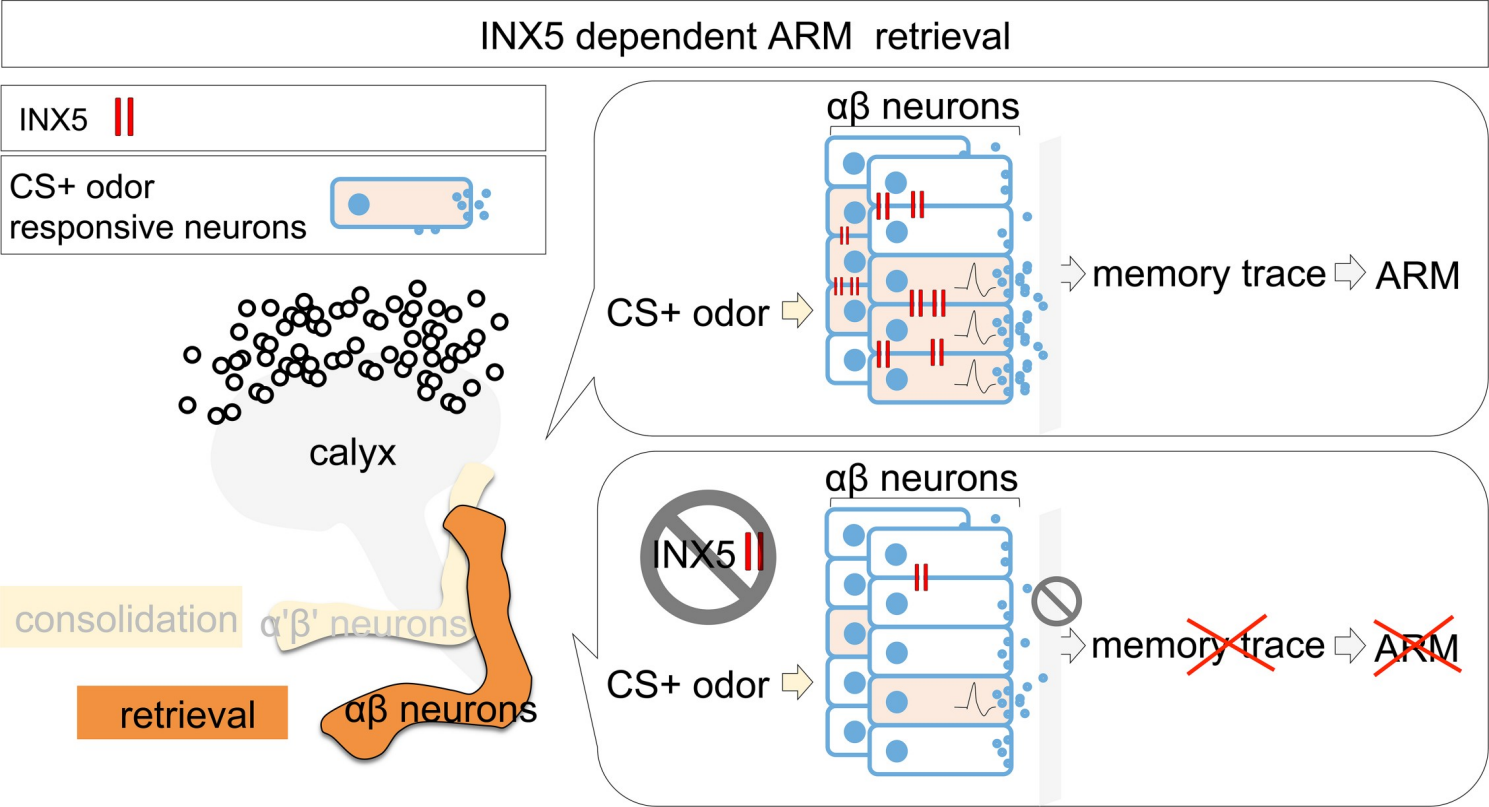
Hypothetical model of the role of INX5 in ARM retrieval. After training, the permeability of gap junction channels composed of INX5 is increased in αβ neurons via an unknown mechanism. During ARM retrieval, INX5 gap junctions propagate the action potential and synchronize the firing of αβ neurons, which contributes to an increase in the proportion of odor responsive αβ neurons to the training odor (CS+ odor). The odor responsive αβ neurons boost the synaptic output strength that induces the relevant branch-specific modification of calcium influx in αβ neurons to the CS+ odor (memory trace). Inhibiting the gap junction channels reduces the proportion of odor responsive αβ neurons to the CS+ odor during memory retrieval, which disrupts branch-specific memory traces and ARM.

To further test the hypothesis that INX5 is involved in ARM retrieval, the calcium indicator GCaMP6 was applied to monitor αβ neuronal activity during ARM retrieval[29]. The preferential expression of INX5 in the somas of MB neurons led us to examine the αβ neuronal activity changes in this region. We first observed that odor/shock association increases the proportion of odor responsive αβ neurons to the conditioned odor (CS+ odor) at 3-hour after a 2-min cold shock given at 2-hour after training (Fig 6A). The increased CS+ odor responsive αβ neurons were diminished by treatment with the gap junction blocker CBX, 10 min before image recording (Fig 6A). To further analyze whether this increased CS+ odor responsive αβ neurons correlate to the memory trace[30, 31], we recorded the neuronal activity in the MB α- and β- lobes respectively. The MB α-lobe branch has been shown to produce training-induced modifications in odor-evoked cellular calcium responses[30]. We visualized the functional responses of naïve flies to different odors, 3-octanol (OCT) or 4-methyl-cyclohexanol (MCH), in the αβ lobes by expressing *UAS-GCaMP6m* under the control of *C739-GAL4*. However, only the MB α-lobe branch exhibited a significantly elevated *GCaMP6* intensity 3 hours after shock/odor association as compared to naïve flies (Fig 6B–6C and S6 Fig). These results indicated that the MB α-lobe branch exhibits a training-induced increase in calcium responses 3 hours after training. The increased calcium response was abrogated by treatment with the gap junction blocker CBX 10 min before image recording, and was recovered after CBX washout (Fig 6B–6C). Although a significant 3-hour memory trace was also observed in α'β' neurons, this phenomenon still occurred with CBX treatment suggesting that the memory trace is independent of the gap junction (S7 Fig). Finally, genetic knockdown of *inx5* in αβ neurons also abolished the increased calcium responses in the MB α-lobe to the training odor at 3-hour after conditioning, which suggests that INX5 gap junctions are required for the branch-specific modification of neuronal responses to the conditioned odor during memory retrieval (Figs 6 and 7).

## Discussion

In fruit flies, two parallel MB circuits, containing αβ and α'β' neurons, are involved in ARM formation. *Radish* expression in αβ neurons is required for partial ARM, whereas *octβ2R* expression in MB α'β' neurons is required for the rest part of ARM, suggesting that two distinct cellular mechanisms regulate ARM in different MB neurons[17, 19, 20, 32]. The *radish* gene encodes a protein with a predicted cAMP-dependent protein kinase phosphorylation site, which can bind Rac1 to regulate the rearrangement of the cytoskeleton and affect synaptic structural morphology[32]. The interaction of RADISH and BRUCHPILOT at the synaptic active zone has been proposed to regulate neurotransmitter release[17], and genetic knockdown of *radish* or *bruchpilot* in αβ neurons disrupts ARM[17, 20]. A recent study indicated that Drk–Drok signaling is essential for ARM formation in αβ neurons, and related to dynamic cytoskeletal changes[33]. In addition, the dopamine type 2 (D2R) and serotonin (5HT1A) receptors in αβ neurons are also critical for ARM formation[34, 35].

The key finding of our study is that the gap junction protein INX5 in αβ neurons is critical for 3-hour ARM retrieval. This conclusion is supported by four independent lines of evidence. First, immunohistochemistry data indicated that INX5 was preferentially expressed in the MB calyxes and somas, and these INX5-positive signals were reduced in *OK107-GAL4* > *UAS-inx5*^*RNAi*^ flies (Fig 2). Second, adult-stage-specific knockdown of *inx5* in αβ neurons impaired ARM (Fig 4). Third, *eNpHR*-mediated inhibition of action potential in αβ neurons during retrieval also impaired ARM (Fig 5). Forth, knockdown of *inx5* in αβ neurons inhibited the training-induced cellular calcium responses in the MB α-lobe region 3 hours after odor/shock association (Fig 6).

Previous studies have concluded that αβ neuronal activity is involved in 3-hour memory retrieval using *shibire*^*ts*^ to transiently block chemical synaptic transmissions via inhibiting neurotransmitter recycling[22, 36]. Three-hour memory is composed of ASM and ARM, each accounting for about half of the memory retention level[3–5]. In our recent study, we further showed that the inhibition of neurotransmitter recycling in αβ neurons during memory retrieval disrupted 3-hour ARM[20]. However, blocking neurotransmitter recycling in αβ neurons during memory acquisition and consolidation did not affect 3-hour ARM (S5A Fig). The function of gap junctions in the electrical synapses is to coordinate the propagation of action potential in neuronal networks[23–27], and *shibire*^*ts*^ cannot block gap junction-mediated electrical synapses. We therefore used *eNpHR* to transiently silence action potential in αβ neurons to confirm the requirement of αβ neuronal activity during the ARM formation process. Our data showed an *eNpHR*-mediated hyperpolarization of αβ neurons during memory retrieval but not acquisition or consolidation, impaired ARM, suggesting that action potential in αβ neurons is required only for ARM retrieval (Fig 5 and S5B Fig). Brain immunostaining data showed that INX5 gap junction proteins are strongly expressed in the calyxes and somas of αβ neurons (Fig 2 and S2 Fig), and knockdown of *inx5* gap junction gene in αβ neurons disrupted ARM (Figs 3 and 4). The expression of gap junction is critical for neuronal functions since it plays a role in the propagation of action potential between adjacent neurons[23–27]. We therefore conclude that the gap junction channels composed of INX5 in αβ neurons are critical for ARM retrieval (Fig 7). A recent study showed that the gap junction protein INX2 regulates calcium transmission across the follicle cells during *Drosophila* oogenesis[37]. In addition, INX1/INX2 induces calcium oscillations in the glial cells of the blood-brain barrier (BBB), enabling signal amplification and synchronization across the BBB in fruit flies[26]. Furthermore, the gap junction protein INX6 is important for promoting synchronous neuronal activity in the dorsal fan-shaped body (dFB) in the fly brain that is critical for the sleep switch[38]. In mammals, most neuronal gap junctions in the brain are composed of Connexin-36 (Cx36) and are involved in synchronizing the hippocampal neuronal oscillatory patterns[39], which is required for emotional memories[8]. Therefore, it is possible that gap junction channels composed of INX5 mediate neuronal activity amplification and synchronization across αβ neurons, boosting the synaptic output strength during ARM retrieval (Fig 7).

By using the newly developed calcium indicator *GCaMP6*[29], we observed the increased proportion of training odor-responsive αβ neurons 3 hours after odor/shock association, and this phenomenon was abolished after treatment with gap junction blocker CBX (Fig 6A). Furthermore, we observed significant enhancement of the training-induced cellular calcium response to the training odor in the MB α-lobe branch 3 hours after odor/shock association (Fig 6B–6C). According to the broad consensus of the field, the memory trace is supposed to be formed in the vertical lobe of the MBs by the activity contingency of MBs and dopaminergic Protocerebral Posterior Lateral 1 (PPL1) neurons, which represent odor and punitive shock, respectively[40–42]. Therefore, it is possible that 3-hour memory trace back propagation of somas’ activity occurs from the MB lobes during memory retrieval. In addition, the branch specific modifications via MB input neurons (e.g., Protocerebral Anterior Medial, PAM) may occur during memory retrieval[43, 44], hence the memory trace was only observed in α-lobe branch but not the β-lobe of MBs (Fig 6B–6C & S6 Fig). This training-induced 3-hour ARM-specific memory trace was eliminated by treatment with the gap junction blocker, CBX, during memory retrieval or by genetic knockdown of *inx5* in αβ neurons. Although a significant 3-hour ARM-specific memory trace was also observed in α'β' neurons, this phenomenon was independent of the gap junction (S7 Fig). From this, we propose that an unknown dynamic mechanism regulates the permeability of gap junction channels composed of INX5 in αβ neurons after training. Recently, cryoelectron microscopy revealed that the structure of *C*.*elegans* INX6 was highly similar to that of the vertebrate gap junction protein Connexin-26 (Cx26)[45]. Connexin properties, such as gating and assembly, can be regulated by phosphorylation[46, 47]. Additionally, the functions of Innexins or Connexins can also be regulated by changes in the intracellular pH and calcium levels[48–50]. Establishing whether the properties of INX5 in the MBs are modified following conditioned training will provide insights into the neuronal mechanisms of ARM.

## Materials and methods

### Fly stocks

Flies were raised on standard cornmeal food media at 25°C and 70% relative humidity under a 12:12-hour light: dark cycle. The “Cantonized” *w*^*1118*^ *w (CS10)* strain was used as a wild-type control. The *MB-GAL80*, *UAS-GCaMP6m*, and *OK107-GAL4* flies were obtained from Bloomington *Drosophila* stock center. The *UAS-eNpHR-YFP; UAS-eNpHR-YFP* fly line was obtained from Ann-Shyn Chiang. The RNAi lines were obtained from the Vienna *Drosophila* RNAi Center or TRiP RNAi fly stocks. All RNAi lines from the Vienna *Drosophila* RNAi Center have been described previously[12]. The *VT30604-GAL4*, *VT44966-GAL4*, *C739-GAL4*, *VT49246-GAL4*, *VT0765-GAL4*, *UAS-shi*^*ts*^, and *tubP-GAL80*^*ts*^ flies have been described[20].

### Whole-mount immunostaining

Brain samples were stained with the mouse 4F3 anti-discs large (DLG) monoclonal antibody (Hybridoma Bank) to label all neuronal synapses, or with a rabbit polyclonal anti-INX5 antibody. The rabbit INX5 antibody was generated by Antibody International, Inc., with an HPLC-purified synthetic peptide, NH_2_– PHFRSSLRRIGEYNEAYAR–COOH, selected from the INX5 sequence. Fixed brain samples were incubated in PBS containing 1% Triton X-100 and 0.25% normal goat serum (PBS-T) with mouse 4F3 anti-DLG antiserum (1:10) or rabbit anti-INX5 (1:1,000) as primary antibodies at 25°C for 1 day. After three washes in PBS-T, the samples were incubated in biotinylated goat anti-mouse or rabbit IgG (1:200; Invitrogen) at 25°C for 1 day. Next, the brain samples were washed and incubated in Alexa Fluor 633 streptavidin (1:500; Invitrogen) at 25°C overnight. After extensive washing, the brain samples were cleared and mounted in FocusClear^T^ (CelExplorer) for confocal imaging.

### Confocal microscopy

Sample brains were imaged under a Zeiss LSM 700 confocal microscope with either a 40× C-Apochromat water-immersion objective lens for whole-brain images (N.A. value, 1.2; working distance, 220 μm) or a 63× glycerol-immersion objective lens for horizontal, sagittal, and frontal cross sections (N.A. value, 1.4; working distance, 170 μm). To overcome the limited field of view, some samples were imaged twice, one for each hemisphere, with overlap in between. We then stitched the two parallel image stacks into a single dataset online with the ZEN software, using the overlapping region to align the two stacks.

### Behavioral assay

Groups of approximately 100 flies were exposed first to one odor (CS+; OCT or MCH) paired with 12 1.5-s pulses of 75-V DC electric shock presented at 5-s interpulse intervals. This was followed by the presentation of a second odor (CS–; MCH or OCT) without electric shock. In the testing phase, the flies were presented with a choice between the CS+ and CS–odors in a T-maze for 2-min. At the end of the 2-min period, the flies in each T-maze arm were trapped, anesthetized, and counted. From the distribution of flies between the 2 arms, the performance index (PI) was calculated as the number of flies avoiding the shock-associated odor (CS+) minus the number avoiding the non-shock-associated odor (CS–), divided by the total number of flies and multiplied by 100. If the flies did not learn, they were distributed equally between the 2 arms; hence, the calculated PI was 0. If all flies avoided the shock-paired odor and were distributed 0:100 between the CS+ and CS–arms in the T-maze, the PI was 100. To assess learning, performance was measured immediately after training. To evaluate intermediate-term memory, testing was performed 3 hours after training. ARM was defined as 3-hour memory after a 2-min cold shock presented at 2-hour post-training (i.e., 1 hour before testing) by placing a plastic vial containing the trained flies in ice water. A brief cold shock completely erases short-term memory and the labile ASM, preserving only ARM. For the adult-stage-specific RNAi-mediated knockdown of *inx5* with *tubP-GAL80*^*ts*^, flies were kept at 18°C until eclosion and then shifted to 30°C for 7 days before training. The 3-hour ARM assay was also performed at 30°C. Control flies were kept at 18°C throughout the experiment. For *eNpHR*-mediated light-inactivation during memory acquisition, a group of approximately 100 flies were put into a custom-made light-delivering electrical shock tube and received electrical shock alternately paired with either OCT or MCH. For *eNpHR*-mediated light-inactivation during memory consolidation, the conditioned flies were put into the LED-embedded tube for 1.5 hours immediately after training. For *eNpHR*-mediated light-inactivation during memory retrieval, the conditioned flies were tested for approach to OCT or MCH in LED-embedded tubes. The light intensity was approximately 9.35 mW/cm^2^, and the wavelength was 590 nm. Flies were fed a standard food medium with or without 100 μM all-trans-retinal (Sigma-Aldrich) for at least 5 days before the experiments.

### GCaMP imaging

Sample preparation for *in vivo* calcium imaging was modified from a previous study[31]. The fly was fixed in a 250-μl pipette tip, a small window was opened on the head capsule using fine tweezers and fixed in place with dental glue. Next, a drop of adult hemolymph-like (AHL) saline (108 mM NaCl, 5 mM KCl, 2 mM CaCl2, 8.2 mM MgCl_2_, 4 mM NaHCO_3_, 1 mM NaH_2_PO_4_, 5 mM trehalose, 10 mM sucrose and 5 mM HEPES [pH 7.5, 265 mOsm]) was added to the window to prevent dehydration. The fly and pipette tip were fixed to a coverslip by tape and time-lapse recording of changes in *GCaMP6m* intensity before and after odor delivery was performed on a Zeiss LSM 700 confocal microscope with a 40X water-immersion objective (W Plan-Apochromat 40× /1.0 DIC M27), a 488-nm excitation laser, and a detector for emissions passing through a 555 nm short-pass filter. An optical slice with a resolution of 512 × 512 pixels was continuously monitored for 60 s at 2 frames per second. Odorants were delivered at 11 s and 29 s in each 60 s trial. To correct the motion artifacts, frames were aligned using a lightweight SIFT-implementation[51]. Response amplitudes were calculated as the mean change in fluorescence (dF/F) in the 0.1–5 s window after stimulus onset. To quantify the numbers of the MB neurons in odor stimulus, the response was judged to be significant if the peak was > 0.2 (dF/F). For the lobe specific memory trace assay, regions of interest (ROI) were manually assigned to anatomically different regions of the MB lobe. To evaluate responses to different odors in flies, we calculated the change in GCaMP6 fluorescence as ΔF (Ft–F_0_)/F_0_. Changes in GCaMP6 fluorescent intensity for the CS+ vs. CS− odors were calculated as log_10_ (ΔF_CS+_/ΔF_CS-_). For the CBX treatment experiments, flies were dissected and immediately placed in a drop of adult AHL saline containing 1 mM CBX for 10 min before image recording. The CBX solution was washed out using standard AHL solution. ΔF/F_0_ intensity maps were generated using ImageJ.

### Quantification of INX5 immunostaining

For the quantification of INX5 protein, fly brains were immunostained with rabbit INX5 antibody (1:1,000) at 25°C for 1 day. After three washes in PBS-T, the samples were incubated in biotinylated goat anti-mouse or rabbit IgG (1:200; Invitrogen) at 25°C for 1 day. The brain samples were then washed and incubated in Alexa Fluor 635 streptavidin (1:500; Invitrogen) at 25°C overnight. After extensive washing, the brain samples were cleared and mounted in FocusClear (CelExplorer). Brain images were obtained using a Zeiss LSM 700 confocal microscope under the same confocal settings for each sample, and the images were further analyzed using ImageJ. Single optical sections were used to calculate the average intensity values per voxel of the INX5 immunopositive signals in the MB calyx, α lobe, α' lobe, β lobe, β' lobe, γ lobe, and protocerebrum bridge (PB). The fluorescent intensity in the PB was used as an adjacent-region control.

### Quantitative PCR (qPCR)

The efficiency of gene inactivation in each *inx*^*RNAi*^ line from TRiP collections was verified with qPCR. Flies for qPCR were generated by crossing *elav-GAL4* virgin flies to either wild-type males or the various *UAS-inx*^*RNAi*^ males. RNA from the isolated heads of adult flies was extracted with TRIzol Reagent (Invitrogen). The extracted RNA was used to synthesize first-strand cDNA with RevertAid First Strand cDNA Synthesis Kit (Thermo Fisher Scientific). RNA expression levels were quantified with SYBR Green PCR Master Mix on a StepOnePlus System (Thermo Fisher Scientific).

### Western blotting

For western blotting, the heads of adult flies were homogenized in lysis buffer (25 mM HEPES [pH 7.5], 100 mM NaCl, 1 mM MgCl_2_, 1 mM CaCl_2_, 0.1% SDS, 0.2% TritonX-100, 0.2% NP-40, 1 mM EDTA, 1 mM EGTA, and protease inhibitor cocktail [Roche, CH]), the lysates were centrifuged at 14,000 rpm for 30 min at 4°C, and the supernatants were collected. Lysate proteins were electrophoresed on an SDS-PAGE and electroblotted onto PVDF membranes. The immobilized proteins were probed with rabbit anti-INX5 (1:20,000) and anti-β-actin (1:10,000) antibodies. The membrane was then incubated with horse radish peroxidase (HRP)-conjugated goat-anti-rabbit IgG secondary antibody (1:10,000). The positive signal was detected with SuperSignal West Pico PLUS Chemiluminescent Substrate (Thermo Fisher Scientific).

### Statistical analysis

Raw data were analyzed parametrically using the Prism 7.0 software (GraphPad). Because of the nature of their mathematical derivation, PIs were distributed normally. Hence, data from more than two groups were evaluated by one-way analysis of variance and Tukey’s multiple-comparisons tests. Data from only two groups were evaluated by the paired t-test. A statistically significant difference was defined as P < 0.05. The data in the bar graphs are presented as means ± SEM.

## Supporting information

S1 Fig Downregulation of INX5 in MBs impairs ARM. (A) Three-hour memory (performance index) in flies carrying one of the eight *UAS-inx*^*RNAi*^ (Transgenic RNAi Project [TRiP] at Harvard Medical School) effectors under the control of the MB-specific driver *OK107-GAL4*. Each value represents the mean ± SEM (n = 8). n.s.: not significant (p > 0.05); *, p < 0.05; one-way analysis of variance (ANOVA) followed by Tukey’s test. The TRiP stock numbers for the *UAS-inx*^*RNAi*^ effectors were as follows: *UAS-inx1*^*RNAi*^(JF02595), *UAS-inx2*^*RNAi*^(JF02446), *UAS-inx3*^*RNAi*^(HM05245), *UAS-inx4*^*RNAi*^(JF02753), *UAS-inx5*^*RNAi*^(JF02877), *UAS-inx6*^*RNAi*^(JF02168), *UAS*-*inx7*^*RNAi*^(JF02066), and *UAS-inx8*^*RNAi*^(JF02604), respectively. The genotypes were as follows: (1) *+/+*, (2) *OK107-GAL4/+*, (3) *UAS-inx*^*RNAi*^(TRiP)*/+*, and (4) *OK107-GAL4/UAS-inx*^*RNAi*^(TRiP). (B) Three-hour ARM test was performed on flies carrying the *OK107-GAL4* driver and *UAS-inx5*^*RNAi*^*(JF02877)* transgene. The flies were trained and tested 3 hours later; the 2-min cold shock was applied at 2 hours after training. Each value represents the mean ± SEM (n = 8). *, p < 0.05; t-test. The genotypes were as follows: (1) *+/+; +/UAS-inx5*^*RNAi*^*(JF02877)*, (2) *+/+; +/UAS-inx5*^*RNAi*^*(JF02877); OK107-GAL4/+*. (C) ASM score (calculated by subtracting the ARM from the total 3-hour memory score) was similar to that in the control group, whereas ARM (light gray) was reduced. This result indicates that ARM is preferentially impaired by INX5 knockdown in MB neurons. The same data as in (A) and (B) are represented. (D) Initial learning was unaffected in the *inx5*-manipulated flies. Each value represents the mean ± SEM (n = 8; p > 0.05, t-test). The genotypes were as follows: (1) *+/+; UAS-inx5*^*RNAi*^*(JF02877)/+*, (2) *+/+; +/UAS-inx5*^*RNAi*^*(JE02877); OK107-GAL4/+*. (E) Quantitative PCR evaluation of the *inx* mRNA levels in the manipulated flies (*elav-GAL4/UAS-inx*^*RNAi*^) relative to those in the control flies (*elav-GAL4/+*). The data were normalized to the relative 60S ribosomal protein L32 (RpL32) level. The qPCR forward and reverse primer sequences were as follows: *inx1*, 5’-ATgCTgggTCgCAATCTTg-3’ and 5-TTggCAAACTCgCTCATCAC-3’; *inx2*, 5’-ATgAgCATAgCgCCCACAA-3’ and 5’-ACggCCACgCCCACTAAT-3’; *inx3*, 5’-ACggCAgATCCgCATgA-3’ and 5’- CATCCggCACACTgACCAT-3’; *inx4*, 5’-CgCgTgggCAACAACA-3’ and 5’-CgTACAgCTCCTCCAgAACTT-3’; *inx5*, 5’-CCTgCCgCTgAACATTCTg-3’ and 5’-gAACCACgCCCAAggAA-3’; *inx6*, 5’-CgTAAAgCCgCTgTCCAACTA-3’ and 5’-AgCgTgAAgATCgggTCgTAT-3’; *inx7*, 5’-TTTTgggCggTCAATTCCT-3’ and 5’-TCggACCACCgATTTTTCA-3’; and *inx8*, 5’-gAAAgATTgTCCAgCCAAAACg-3’ and 5’-TTACggTgTCCgCAACAAgA-3’.
(TIF)

S2 Fig Quantitative INX5 immunostaining in each MB sub-compartment. Single optical sections of confocal images of fly brains immunostained for INX5. All images were taken with the same settings. The average intensity values per voxel were calculated in the MB calyx, α lobe, α' lobe, β lobe, β' lobe, and γ lobe. The protocerebrum bridge (PB) was used as an adjacent control region. The scale bars represent 50 μm. Each value represents the mean ± SEM (n = 10).
(TIF)

S3 Fig Downregulation of INX5 in MBON-β2β' 2a neurons does not impair ARM. Three-hour ARM was tested in flies carrying the *VT0765-GAL4* driver and a *UAS-inx*^*RNAi*^ transgene (VDRC). The flies were trained and tested at 3-hour after training; the 2-min cold shock was applied at 2-hour after training. Each value represents the mean ± SEM (n = 6–10; p > 0.05, ANOVA). The genotypes were as follows: (1) *+/+; VT0765-GAL4/+*, (2) *UAS-inx*^*RNAi*^(VDRC)*/+*, and (3) *OK107-GAL4/UAS-inx*^*RNAi*^(VDRC).
(TIF)

S4 Fig Knockdown *inx5* does not affect gross morphologies of the MB structures. Gross morphologies of the MB structures (green) in control flies (A1-C1) or flies with constitutive expression of the indicated *inx5*^*RNAi*^ transgene driven by *OK107-GAL4* (A2, A3), *VT49246-GAL4* (B2, B3), and *C739-GAL4* (C2, C3). Brain structures were counterstained with DLG antibody (red). The genotypes were as follows: (A1) *+/UAS-mCD8*::*GFP; +/+; OK107-GAL4/+*, (A2) *+/UAS-mCD8*::*GFP; UAS-inx5*^*RNAi*^*(v6950)/+; OK107-GAL4/+*, (A3) *+/UAS-mCD8-GFP; +/UAS-inx5*^*RNAi*^*(JE02877); OK107-GAL4/+*, (B1) *+/UAS-mCD8*::*GFP; VT49246-GAL4/+*, (B2) *+/UAS-mCD8*::*GFP; VT49246-GAL4/UAS-inx5*^*RNAi*^*(v6950)*, (B3) *+/UAS-mCD8-GFP; VT49246-GAL4/UAS-inx5*^*RNAi*^*(JE02877)*, (C1) *C739-GAL4/UAS-mCD8-GFP; +/+*, (C2) *C739-GAL4/UAS-mCD8*::*GFP; +/UAS-inx5*^*RNAi*^*(v6950)*, and (C3) *C739-GAL4/UAS-mCD8-GFP; +/UAS-inx5*^*RNAi*^*(JE02877)*. The scale bars represent 20 μm.
(TIF)

S5 Fig The activity in αβ neurons is required for ARM retrieval. (A) Blocking neurotransmission in αβ neurons by *shi*^*ts*^ during acquisition and consolidation did not affect 3-hour ARM. Neurotransmission was blocked by keeping *shi*^*ts*^ flies at a restrictive temperature (32°C) during training and for 1.5 hours post-training. Cold shock was applied 2-hour after training. Each value represents the mean ± SEM (n = 14). n.s.: not significant (p > 0.05); ANOVA. The genotypes were as follows: (1) *+/+; +/+*, (2) *C739-GAL4/+; +/+*, (3) *+/+; +/UAS-shi*^*ts*^, (4) *C739-GAL4/+; +/UAS-shi*^*ts*^. (B) *eNpHR*-mediated hyperpolarization of αβ neurons during memory retrieval (test) impaired 3-hour ARM. Each value represents the mean ± SEM (n = 6–10). *, p < 0.05; t-test. The genotypes were as follows: (1) *+/UAS-eNpHR-YFP; +/UAS-eNpHR-YFP*, (2) *+/UAS-eNpHR-YFP; VT49246-GAL4/UAS-eNpHR-YFP*.
(TIF)

S6 Fig MB β-lobe branch does not form 3-hour ARM-specific memory trace. (A) Three-hour memory trace was not observed in the MB β-lobe branch. GCaMP6 responses to the training odor (OCT-trained flies: top row; MCH-trained flies: middle row) in the β-lobe axonal branch at 3-hour after training with a 2-min cold shock given at 2-hour postconditioning. (B) Quantification of the GCaMP6 responses to the training odor (CS+) relative to the non-training odor (CS-) in the β-lobes in OCT-trained (left two bars) or MCH-trained (right two bars) flies. Recordings were made in the β-lobe tips. The Log ratios of the CS+ response to the CS- response were calculated using the peak response amplitudes. Each value represents the mean ± SEM (n = 14). n.s.: not significant (p > 0.05); t-test. Genotype: *C739-GAL4*/*UAS-GCaMP6m; +/+*.
(TIF)

S7 Fig 3-hour memory trace in α'β' neurons is gap junction independent. (A) Odor/shock paired training (control group) induced an increase in the GCaMP6 responses in the α'-lobe axonal branch of the MB neurons to the training odor [OCT-trained flies: OCT (CS+), MCH-trained flies: MCH (CS+)], and the increase also occurs with CBX treatment. The GCaMP6 responses were recorded 3 hours after training with a 2-min cold shock given at 2-hour postconditioning. (B) Quantification of the enhanced GCaMP6 responses to the training odor (CS+) relative to the non-training odor (CS-) in the α'-lobe in OCT-trained (left panel) or MCH-trained (right panel) flies. The recordings were performed in the α'-lobe tips. The Log ratios of the CS+ response to the CS- response were calculated using the peak response amplitudes. Each value represents the mean ± SEM (n = 6 for each bar). *, p < 0.05; one-way analysis of variance followed by Tukey’s test. Genotype: *+*/*UAS-GCaMP6m; VT30604-GAL4/+*.
(TIF)

## References

[1] Tully T, Quinn WG. Classical conditioning and retention in normal and mutant Drosophila melanogaster. J Comp Physiol A. 1985;157:263–77. 10.1007/BF01350033 . 3939242

[2] Aso Y, Grubel K, Busch S, Friedrich AB, Siwanowicz I, Tanimoto H. The mushroom body of adult Drosophila characterized by GAL4 drivers. J Neurogenet. 2009;23:156–72. 10.1080/01677060802471718 . 19140035

[3] Quinn WG, Dudai Y. Memory phases in Drosophila. Nature. 1976;262:576–7. . 822344 10.1038/262576a0

[4] Margulies C, Tully T, Dubnau J. Deconstructing memory in Drosophila. Curr Biol. 2005;15:R700–13. 10.1016/j.cub.2005.08.024 . 16139203 PMC3044934

[5] Horiuchi J, Yamazaki D, Naganos S, Aigaki T, Saitoe M. Protein kinase A inhibits a consolidated form of memory in Drosophila. Proc Natl Acad Sci U S A. 2008;105:20976–81 10.1073/pnas.0810119105 19075226 PMC2634933

[6] Hervé J-C, Derangeon M. Gap-junction-mediated cell-to-cell communication. Cell Tissue Res. 2013;352:21–31. 10.1007/s00441-012-1485-6 22940728

[7] Beheshti S, Eivani M, Moshtaghian J. Gap junctions of the hippocampal CA1 area are crucial for memory consolidation. Physiol Pharmacol. 2015;19(3):177–84.

[8] Bissiere S, Zelikowsky M, Ponnusamy R, Jacobs NS, Blair HT, Fanselow MS. Electrical synapses control hippocampal contributions to fear learning and memory. Science. 2011;331:87–91. 10.1126/science.1193785 PMC4276370. 21212357 PMC4276370

[9] Sohl G, Willecke K. An update on connexin genes and their nomenclature in mouse and man. Cell Commun Adhes. 2003;10:173–80. 10.1080/cac.10.4-6.173.180 . 14681012

[10] Paul DL. Molecular cloning of cDNA for rat liver gap junction protein. J Cell Biol. 1986;103:123–34. 10.1083/jcb.103.1.123 3013898 PMC2113807

[11] Phelan P, Starich TA. Innexins get into the gap. Bioessays. 2001;23:388–96. 10.1002/bies.1057 . 11340620

[12] Wu CL, Shih MF, Lai JS, Yang HT, Turner GC, Chen L, et al Heterotypic gap junctions between two neurons in the Drosophila brain are critical for memory. Curr Biol. 2011;21:848–54. 10.1016/j.cub.2011.02.041 . 21530256

[13] Liu Q, Yang X, Tian J, Gao Z, Wang M, Li Y, et al Gap junction networks in mushroom bodies participate in visual learning and memory in. Elife. 2016;5 10.7554/eLife.13238 . 27218450 PMC4909397

[14] Gradinaru V, Thompson KR, Deisseroth K. eNpHR: a Natronomonas halorhodopsin enhanced for optogenetic applications. Brain Cell Biol. 2008;36:129–39. 10.1007/s11068-008-9027-6 . 18677566 PMC2588488

[15] Dietzl G, Chen D, Schnorrer F, Su K-C, Barinova Y, Fellner M, et al A genome-wide transgenic RNAi library for conditional gene inactivation in Drosophila. Nature. 2007;448:151–6. 10.1038/nature05954 17625558

[16] Ni J-Q, Liu L-P, Binari R, Hardy R, Shim H-S, Cavallaro A, et al A Drosophila Resource of Transgenic RNAi Lines for Neurogenetics. Genetics. 2009;182:1089–100. 10.1534/genetics.109.103630 19487563 PMC2728850

[17] Knapek S, Sigrist S, Tanimoto H. Bruchpilot, a synaptic active zone protein for anesthesia-resistant memory. J Neurosci. 2011;31:3453–8. 10.1523/JNEUROSCI.2585-10.2011 . 21368057 PMC6623931

[18] Scheunemann L, Jost E, Richlitzki A, Day JP, Sebastian S, Thum AS, et al Consolidated and labile odor memory are separately encoded within the Drosophila brain. J Neurosci. 2012;32:17163–71. 10.1523/JNEUROSCI.3286-12.2012 . 23197709 PMC6621839

[19] Wu CL, Shih MF, Lee PT, Chiang AS. An octopamine-mushroom body circuit modulates the formation of anesthesia-resistant memory in Drosophila. Curr Biol. 2013;23:2346–54 10.1016/j.cub.2013.09.056 . 24239122

[20] Yang CH, Shih MF, Chang CC, Chiang MH, Shih HW, Tsai YL, et al Additive expression of consolidated memory through Drosophila mushroom body subsets. PLoS Genet. 2016;12:e1006061 10.1371/journal.pgen.1006061 . 27195782 PMC4873240

[21] Stebbings LA, Todman MG, Phillips R, Greer CE, Tam J, Phelan P, et al Gap junctions in Drosophila: developmental expression of the entire innexin gene family. Mech Dev. 2002;113:197–205. 10.1016/S0925-4773(02)00025-4 . 11960713

[22] Krashes MJ, Keene AC, Leung B, Armstrong JD, Waddell S. Sequential use of mushroom body neuron subsets during Drosophila odor memory processing. Neuron. 2007;53:103–15. 10.1016/j.neuron.2006.11.021 . 17196534 PMC1828290

[23] Coulon P, Landisman CE. The Potential Role of Gap Junctional Plasticity in the Regulation of State. Neuron. 2017;93(6):1275–95. 10.1016/j.neuron.2017.02.041 28334604

[24] Pereda AE, Curti S, Hoge G, Cachope R, Flores CE, Rash JE. Gap junction-mediated electrical transmission: Regulatory mechanisms and plasticity. Biochim Biophys Acta. 2013;1828:134–46. 10.1016/j.bbamem.2012.05.026 22659675 PMC3437247

[25] Phelan P, Goulding LA, Tam JLY, Allen MJ, Dawber RJ, Davies JA, et al Molecular Mechanism of Rectification at Identified Electrical Synapses in the Drosophila Giant Fiber System. Curr Biol. 2008;18:1955–60. 10.1016/j.cub.2008.10.067 19084406 PMC2663713

[26] Spéder P, Brand AH. Gap junction proteins in the blood-brain barrier control nutrient-dependent reactivation of Drosophila neural stem cells. Dev Cell. 2014;30:309–21. 10.1016/j.devcel.2014.05.021 . 25065772 PMC4139190

[27] Thomas JB, Wyman RJ. Mutations altering synaptic connectivity between identified neurons in Drosophila. J Neurosci. 1984;4(2):530–8. 10.1523/JNEUROSCI.04-02-00530.1984 6699687 PMC6564902

[28] Turrel O, Goguel V, Preat T. Amnesiac is required in the adult mushroom body for memory formation. J Neurosci. 2018;38(43):9202 10.1523/JNEUROSCI.0876-18.2018 30201766 PMC6705992

[29] Chen T-W, Wardill TJ, Sun Y, Pulver SR, Renninger SL, Baohan A, et al Ultra-sensitive fluorescent proteins for imaging neuronal activity. Nature. 2013;499:295–300. 10.1038/nature12354 PMC3777791. 23868258 PMC3777791

[30] Yu D, Akalal DB, Davis RL. Drosophila alpha/beta mushroom body neurons form a branch-specific, long-term cellular memory trace after spaced olfactory conditioning. Neuron. 2006;52:845–55. 10.1016/j.neuron.2006.10.030 17145505 PMC1779901

[31] Wang Y, Mamiya A, Chiang AS, Zhong Y. Imaging of an early memory trace in the Drosophila mushroom body. J Neurosci. 2008;28:4368–76. 10.1523/JNEUROSCI.2958-07.2008 . 18434515 PMC3413309

[32] Folkers E, Waddell S, Quinn WG. The Drosophila radish gene encodes a protein required for anesthesia-resistant memory. Proc Natl Acad Sci U S A. 2006;103:17496–500. 10.1073/pnas.0608377103 . 17088531 PMC1634833

[33] Kotoula V, Moressis A, Semelidou O, Skoulakis EMC. Drk-mediated signaling to Rho kinase is required for anesthesia-resistant memory in Drosophila. Proc Natl Acad Sci U S A. 2017;114:10984–9. 10.1073/pnas.1704835114 28973902 PMC5642687

[34] Lee P-T, Lin H-W, Chang Y-H, Fu T-F, Dubnau J, Hirsh J, et al Serotonin-mushroom body circuit modulating the formation of anesthesia-resistant memory in Drosophila. Proc Natl Acad Sci U S A 2011;108:13794 10.1073/pnas.1019483108 21808003 PMC3158232

[35] Scholz-Kornehl S, Schwärzel M. Circuit analysis of a Drosophila dopamine type 2 receptor that supports anesthesia-resistant memory. J Neurosci. 2016;36:7936–45. 10.1523/JNEUROSCI.4475-15.2016 27466338 PMC6601883

[36] McGuire SE, Le PT, Davis RL. The role of Drosophila mushroom body signaling in olfactory memory. Science. 2001;293:1330–3. 10.1126/science.1062622 . 11397912

[37] Sahu A, Ghosh R, Deshpande G, Prasad M. A gap junction protein, inx2, modulates calcium flux to specify border cell fate during Drosophila oogenesis. PLoS Genet. 2017;13:e1006542 10.1371/journal.pgen.1006542 . 28114410 PMC5256874

[38] Troup M, Yap MHW, Rohrscheib C, Grabowska MJ, Ertekin D, Randeniya R, et al Acute control of the sleep switch in Drosophila reveals a role for gap junctions in regulating behavioral responsiveness. Elife 2018;7:e37105 10.7554/eLife.37105 30109983 PMC6117154

[39] Posłuszny A. The contribution of electrical synapses to field potential oscillations in the hippocampal formation. Front Neural Circuits. 2014;8:32 10.3389/fncir.2014.00032 PMC3982077. 24772068 PMC3982077

[40] Aso Y, Herb A, Ogueta M, Siwanowicz I, Templier T, Friedrich AB, et al Three dopamine pathways induce aversive odor memories with different stability. PLoS Genet. 2012;8(7):e1002768 10.1371/journal.pgen.1002768 22807684 PMC3395599

[41] Waddell S. Dopamine reveals neural circuit mechanisms of fly memory. Trends Neurosci. 2010;33(10):457–64. 10.1016/j.tins.2010.07.001 20701984 PMC2947577

[42] Claridge-Chang A, Roorda RD, Vrontou E, Sjulson L, Li H, Hirsh J, et al Writing memories with light-addressable reinforcement circuitry. Cell. 2009;139(2):405–15. 10.1016/j.cell.2009.08.034 19837039 PMC3920284

[43] Cohn R, Morantte I, Ruta V. Coordinated and compartmentalized neuromodulation shapes sensory processing in Drosophila. Cell. 2015;163(7):1742–55. 10.1016/j.cell.2015.11.019 26687359 PMC4732734

[44] Aso Y, Hattori D, Yu Y, Johnston RM, Iyer NA, Ngo T-TB, et al The neuronal architecture of the mushroom body provides a logic for associative learning. Elife. 2014;3:e04577 10.7554/eLife.04577 25535793 PMC4273437

[45] Oshima A, Matsuzawa T, Murata K, Tani K, Fujiyoshi Y. Hexadecameric structure of an invertebrate gap junction channel. J Mol Biol. 2016;428:1227–36. 10.1016/j.jmb.2016.02.011 26883891

[46] Laird DW. Connexin phosphorylation as a regulatory event linked to gap junction internalization and degradation. Biochim Biophys Acta. 2005;1711:172–82. 10.1016/j.bbamem.2004.09.009 . 15955302

[47] Moreno AP. Connexin phosphorylation as a regulatory event linked to channel gating. Biochim Biophys Acta. 2005;1711:164–71. 10.1016/j.bbamem.2005.02.016 . 15955301

[48] LW R., Birgit R. Calcium in (junctional) intercellular communication and a thought on its behavior in intracellular communication. Ann N Y Acad Sci. 1978;307:285–307. 10.1111/j.1749-6632.1978.tb41958.x 360941

[49] Spray DC, Bennett MVL. Physiology and pharmacology of gap junctions. Annu Rev Physiol. 1985;47:281–303. 10.1146/annurev.ph.47.030185.001433 2859833

[50] Lampe PD, Lau AF. The effects of connexin phosphorylation on gap junctional communication. Int J Biochem Cell Biol. 2004;36:1171–86. 10.1016/S1357-2725(03)00264-4 15109565 PMC2878204

[51] Lowe DG. Distinctive Image Features from Scale-Invariant Keypoints. Int J Comput Vision. 2004;60:91–110. 10.1023/B:VISI.0000029664.99615.94

