# Supplementary figures and images for: Electrical synapses between mushroom body neurons are critical for consolidated memory retrieval in Drosophila
Source: PLoS Genet. 2019 May 9;15(5):e1008153. doi: 10.1371/journal.pgen.1008153 (PMC6529013; doi:10.1371/journal.pgen.1008153)

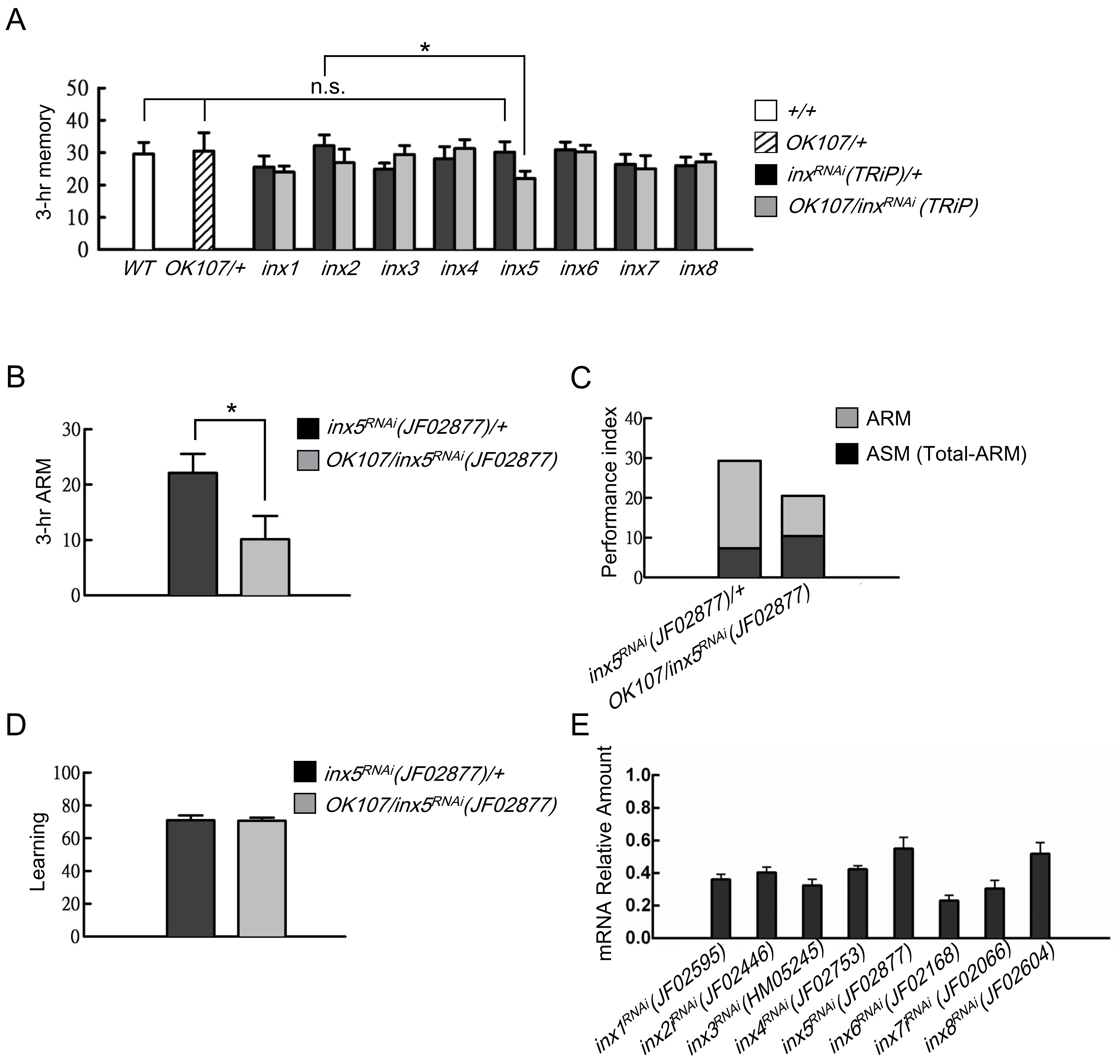

Supplement: S1 Fig — (A) Three-hour memory (performance index) in flies carrying one of the eight UAS-inxRNAi (Transgenic RNAi Project [TRiP] at Harvard Medical School) effectors under the control of the MB-specific driver OK107-GAL4. Each value represents the mean ± SEM (n = 8). n.s.: not significant (p > 0.05); *, p < 0.05; one-way analysis of variance (ANOVA) followed by Tukey’s test. The TRiP stock numbers for the UAS-inxRNAi effectors were as follows: UAS-inx1RNAi(JF02595), UAS-inx2RNAi(JF02446), UAS-inx3RNAi(HM05245), UAS-inx4RNAi(JF02753), UAS-inx5RNAi(JF02877), UAS-inx6RNAi(JF02168), UAS-inx7RNAi(JF02066), and UAS-inx8RNAi(JF02604), respectively. The genotypes were as follows: (1) +/+, (2) OK107-GAL4/+, (3) UAS-inxRNAi(TRiP)/+, and (4) OK107-GAL4/UAS-inxRNAi(TRiP). (B) Three-hour ARM test was performed on flies carrying the OK107-GAL4 driver and UAS-inx5RNAi(JF02877) transgene. The flies were trained and tested 3 hours later; the 2-min cold shock was applied at 2 hours after training. Each value represents the mean ± SEM (n = 8). *, p < 0.05; t-test. The genotypes were as follows: (1) +/+; +/UAS-inx5RNAi(JF02877), (2) +/+; +/UAS-inx5RNAi(JF02877); OK107-GAL4/+. (C) ASM score (calculated by subtracting the ARM from the total 3-hour memory score) was similar to that in the control group, whereas ARM (light gray) was reduced. This result indicates that ARM is preferentially impaired by INX5 knockdown in MB neurons. The same data as in (A) and (B) are represented. (D) Initial learning was unaffected in the inx5-manipulated flies. Each value represents the mean ± SEM (n = 8; p > 0.05, t-test). The genotypes were as follows: (1) +/+; UAS-inx5RNAi(JF02877)/+, (2) +/+; +/UAS-inx5RNAi(JE02877); OK107-GAL4/+. (E) Quantitative PCR evaluation of the inx mRNA levels in the manipulated flies (elav-GAL4/UAS-inxRNAi) relative to those in the control flies (elav-GAL4/+). The data were normalized to the relative 60S ribosomal protein L32 (RpL32) level. The qPCR forward and reverse primer sequence [file pgen.1008153.s001.tif]

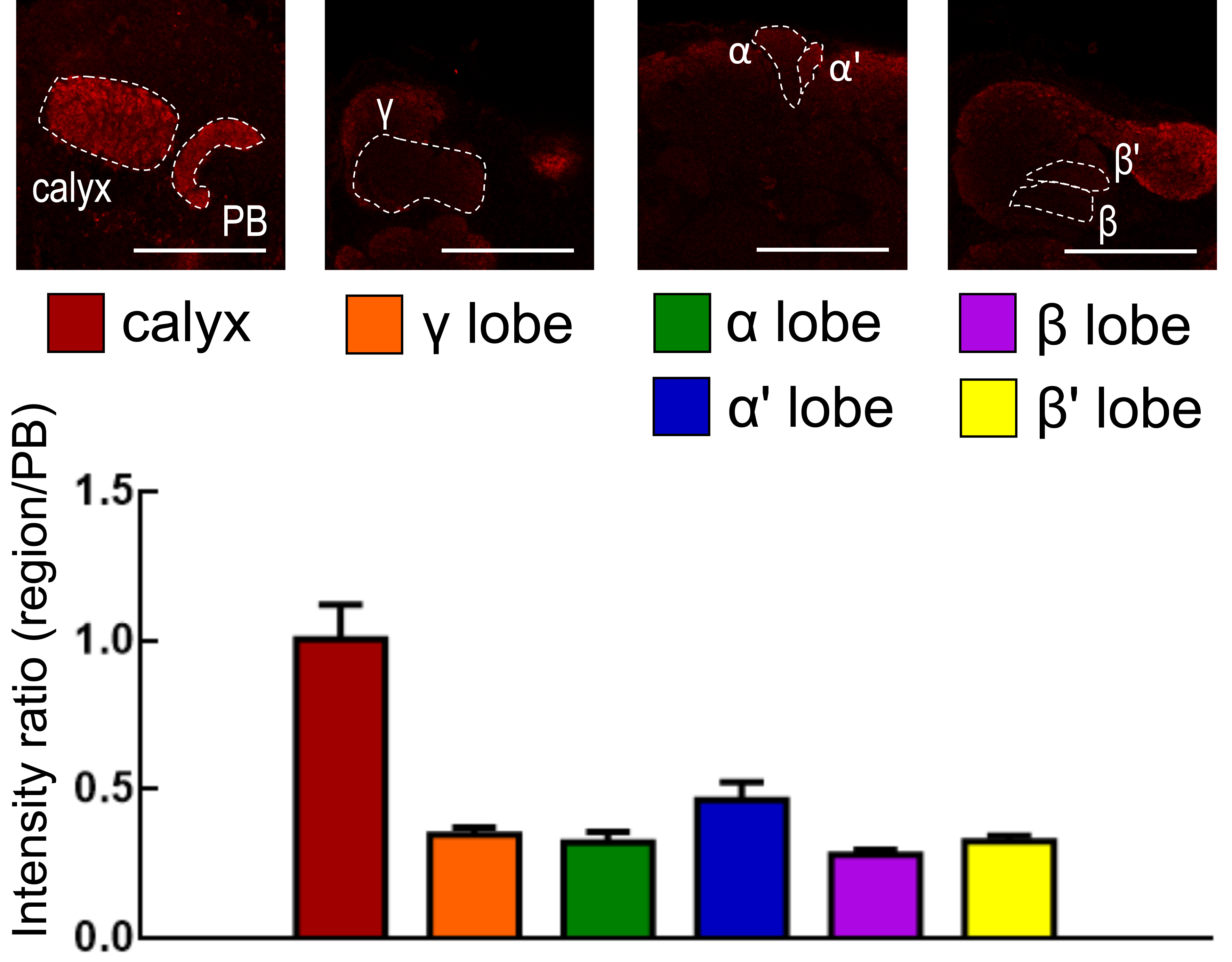

Supplement: S2 Fig — Single optical sections of confocal images of fly brains immunostained for INX5. All images were taken with the same settings. The average intensity values per voxel were calculated in the MB calyx, α lobe, α' lobe, β lobe, β' lobe, and γ lobe. The protocerebrum bridge (PB) was used as an adjacent control region. The scale bars represent 50 μm. Each value represents the mean ± SEM (n = 10). (TIF) [file pgen.1008153.s002.tif]

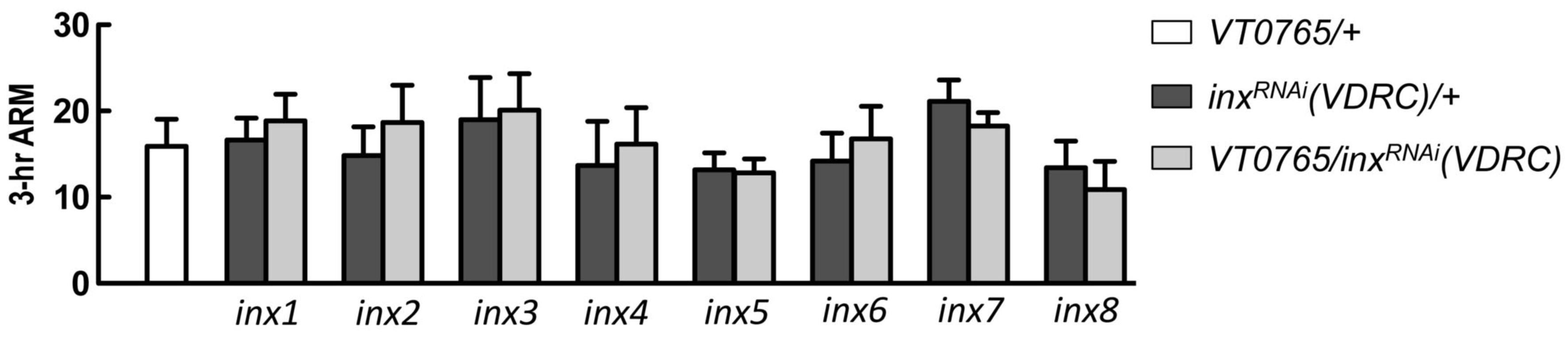

Supplement: S3 Fig — Three-hour ARM was tested in flies carrying the VT0765-GAL4 driver and a UAS-inxRNAi transgene (VDRC). The flies were trained and tested at 3-hour after training; the 2-min cold shock was applied at 2-hour after training. Each value represents the mean ± SEM (n = 6–10; p > 0.05, ANOVA). The genotypes were as follows: (1) +/+; VT0765-GAL4/+, (2) UAS-inxRNAi(VDRC)/+, and (3) OK107-GAL4/UAS-inxRNAi(VDRC). (TIF) [file pgen.1008153.s003.tif]

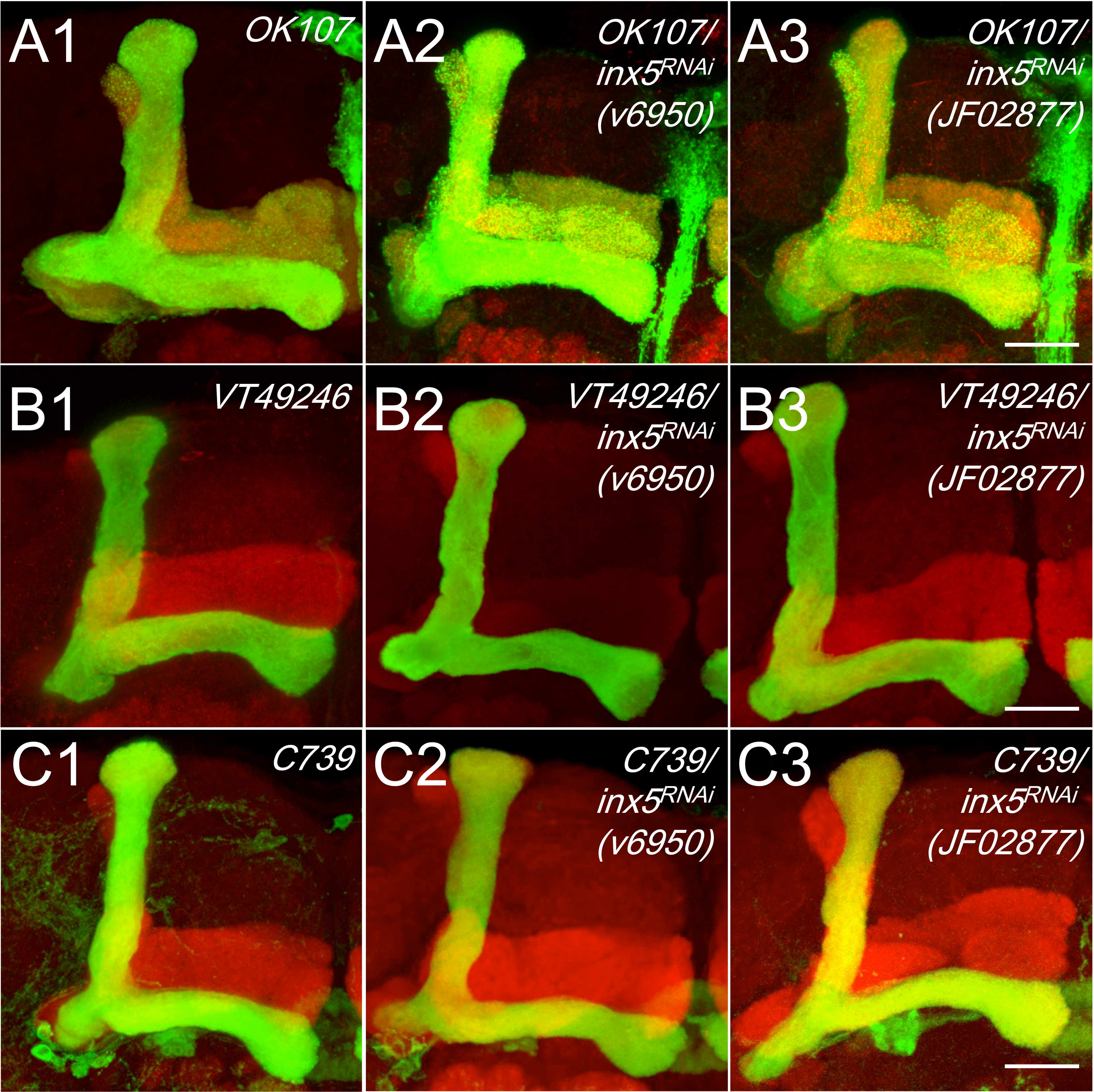

Supplement: S4 Fig — Gross morphologies of the MB structures (green) in control flies (A1-C1) or flies with constitutive expression of the indicated inx5RNAi transgene driven by OK107-GAL4 (A2, A3), VT49246-GAL4 (B2, B3), and C739-GAL4 (C2, C3). Brain structures were counterstained with DLG antibody (red). The genotypes were as follows: (A1) +/UAS-mCD8::GFP; +/+; OK107-GAL4/+, (A2) +/UAS-mCD8::GFP; UAS-inx5RNAi(v6950)/+; OK107-GAL4/+, (A3) +/UAS-mCD8-GFP; +/UAS-inx5RNAi(JE02877); OK107-GAL4/+, (B1) +/UAS-mCD8::GFP; VT49246-GAL4/+, (B2) +/UAS-mCD8::GFP; VT49246-GAL4/UAS-inx5RNAi(v6950), (B3) +/UAS-mCD8-GFP; VT49246-GAL4/UAS-inx5RNAi(JE02877), (C1) C739-GAL4/UAS-mCD8-GFP; +/+, (C2) C739-GAL4/UAS-mCD8::GFP; +/UAS-inx5RNAi(v6950), and (C3) C739-GAL4/UAS-mCD8-GFP; +/UAS-inx5RNAi(JE02877). The scale bars represent 20 μm. (TIF) [file pgen.1008153.s004.tif]

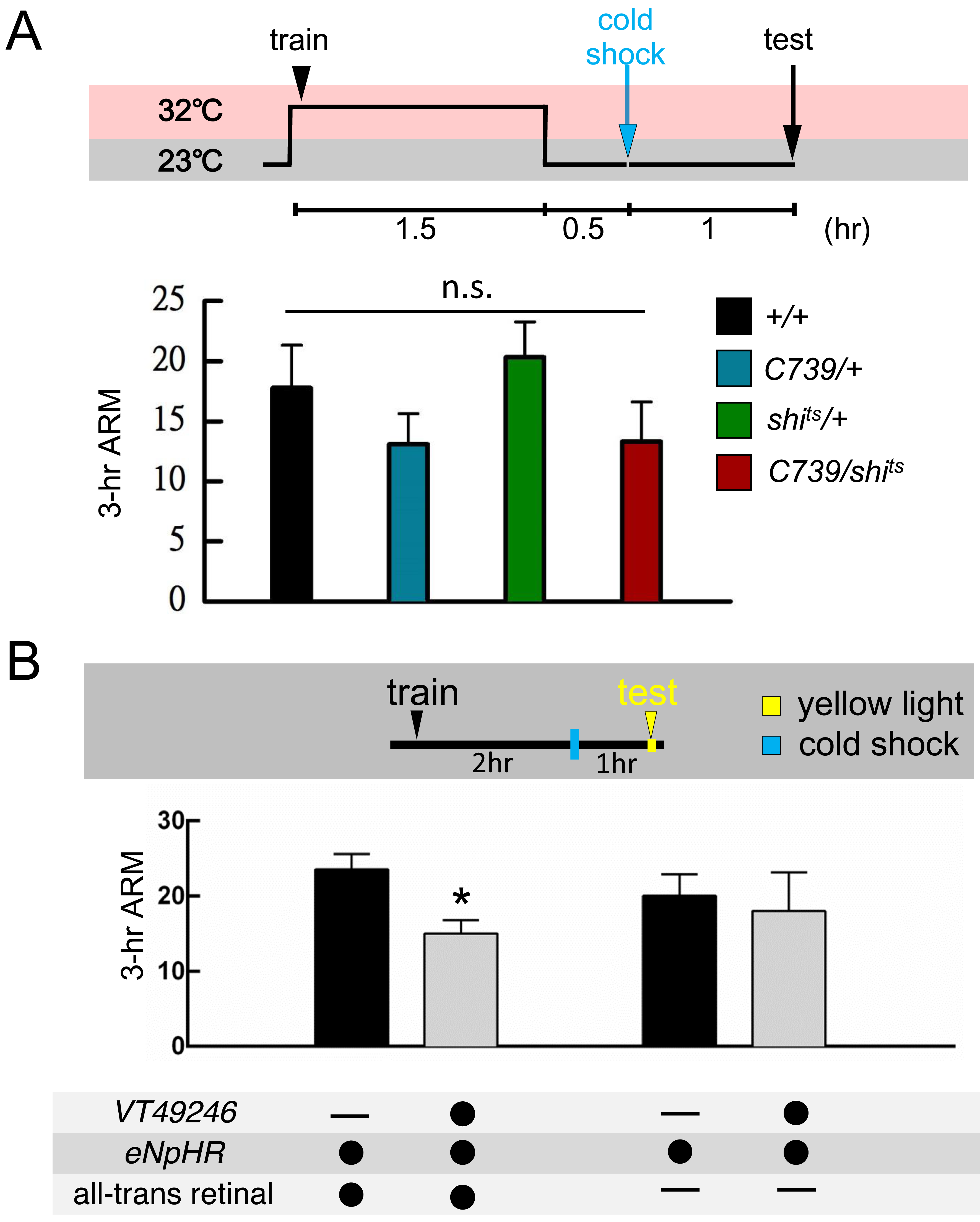

Supplement: S5 Fig — (A) Blocking neurotransmission in αβ neurons by shits during acquisition and consolidation did not affect 3-hour ARM. Neurotransmission was blocked by keeping shits flies at a restrictive temperature (32°C) during training and for 1.5 hours post-training. Cold shock was applied 2-hour after training. Each value represents the mean ± SEM (n = 14). n.s.: not significant (p > 0.05); ANOVA. The genotypes were as follows: (1) +/+; +/+, (2) C739-GAL4/+; +/+, (3) +/+; +/UAS-shits, (4) C739-GAL4/+; +/UAS-shits. (B) eNpHR-mediated hyperpolarization of αβ neurons during memory retrieval (test) impaired 3-hour ARM. Each value represents the mean ± SEM (n = 6–10). *, p < 0.05; t-test. The genotypes were as follows: (1) +/UAS-eNpHR-YFP; +/UAS-eNpHR-YFP, (2) +/UAS-eNpHR-YFP; VT49246-GAL4/UAS-eNpHR-YFP. (TIF) [file pgen.1008153.s005.tif]

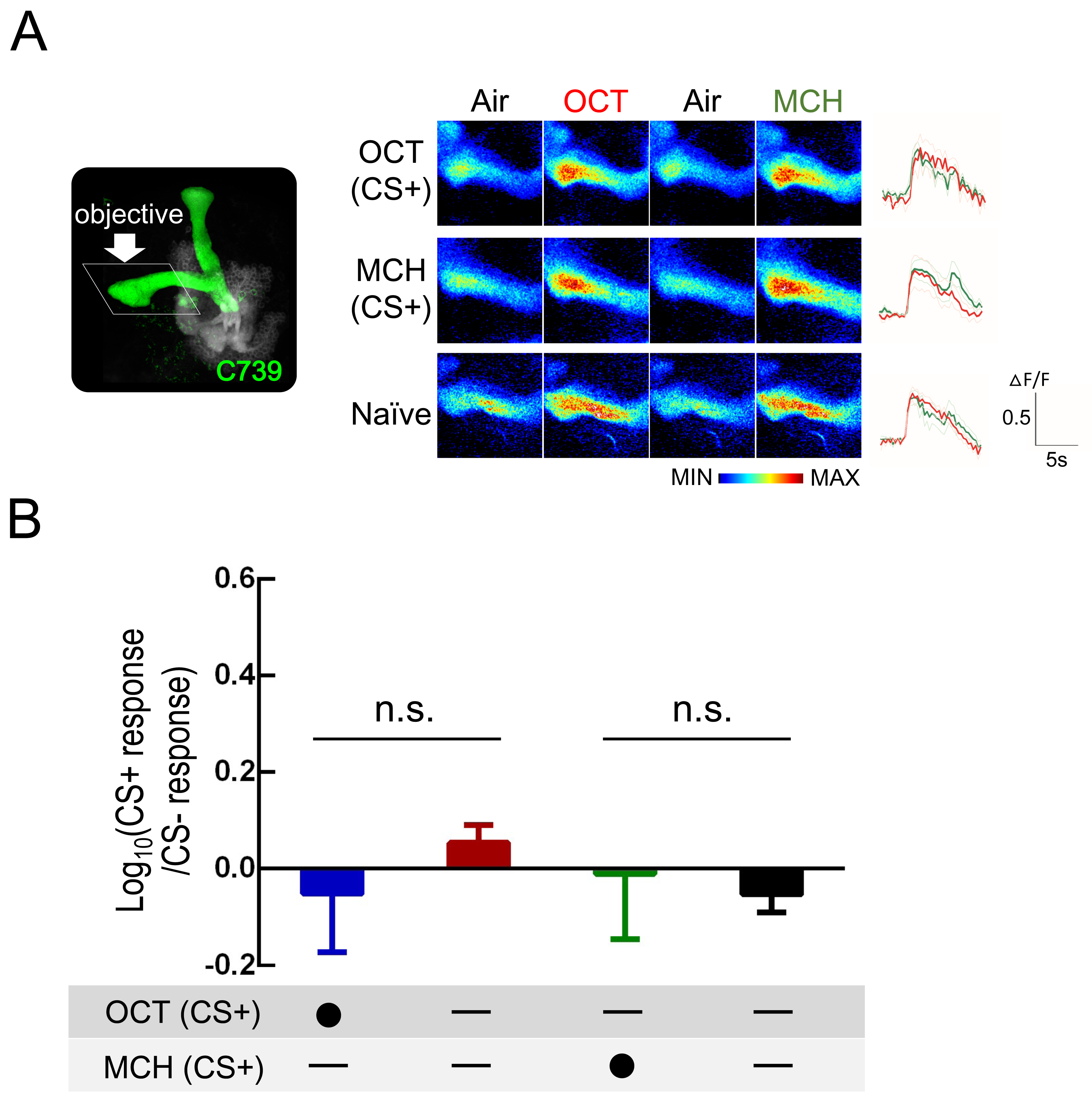

Supplement: S6 Fig — (A) Three-hour memory trace was not observed in the MB β-lobe branch. GCaMP6 responses to the training odor (OCT-trained flies: top row; MCH-trained flies: middle row) in the β-lobe axonal branch at 3-hour after training with a 2-min cold shock given at 2-hour postconditioning. (B) Quantification of the GCaMP6 responses to the training odor (CS+) relative to the non-training odor (CS-) in the β-lobes in OCT-trained (left two bars) or MCH-trained (right two bars) flies. Recordings were made in the β-lobe tips. The Log ratios of the CS+ response to the CS- response were calculated using the peak response amplitudes. Each value represents the mean ± SEM (n = 14). n.s.: not significant (p > 0.05); t-test. Genotype: C739-GAL4/UAS-GCaMP6m; +/+. (TIF) [file pgen.1008153.s006.tif]

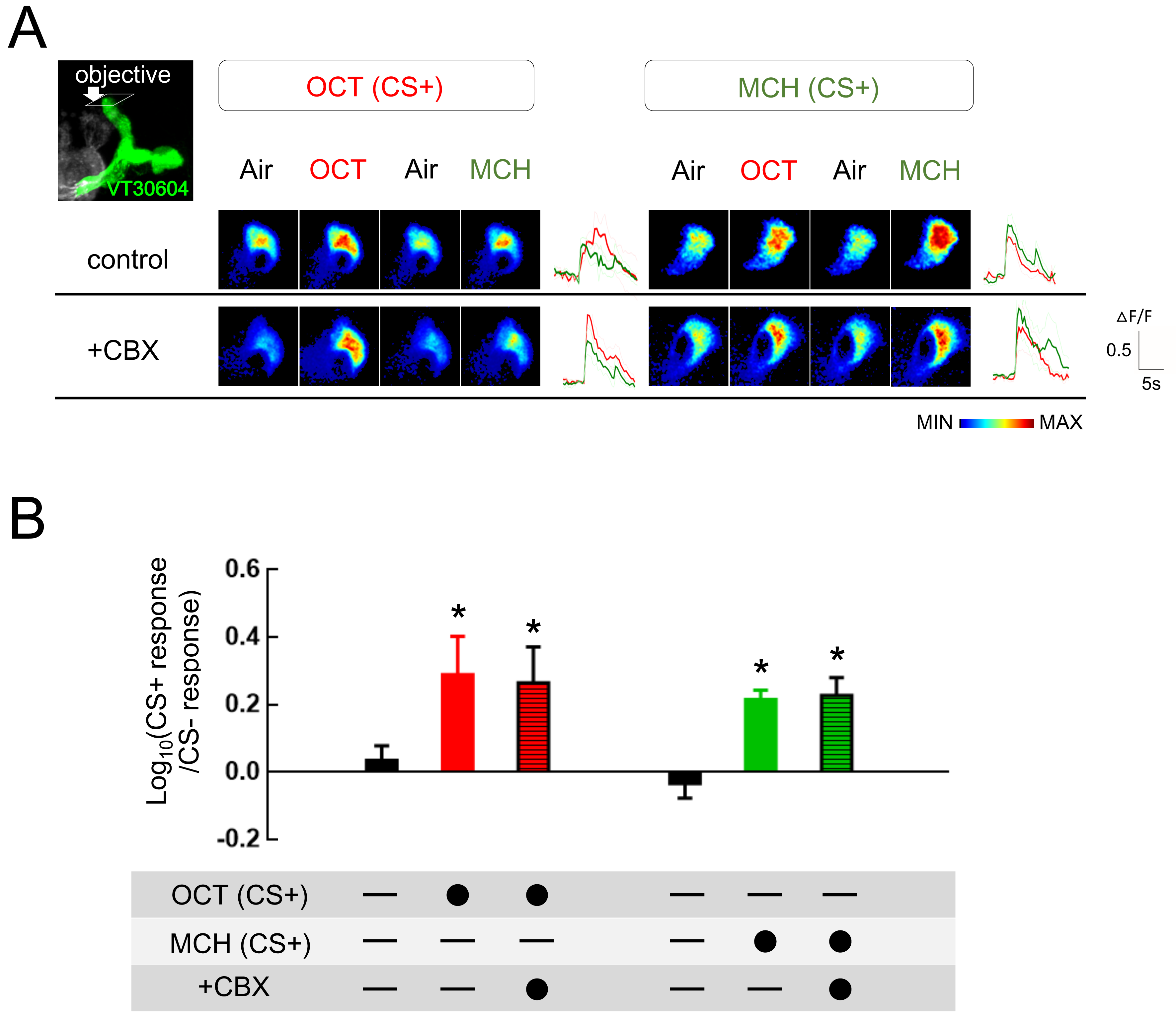

Supplement: S7 Fig — (A) Odor/shock paired training (control group) induced an increase in the GCaMP6 responses in the α'-lobe axonal branch of the MB neurons to the training odor [OCT-trained flies: OCT (CS+), MCH-trained flies: MCH (CS+)], and the increase also occurs with CBX treatment. The GCaMP6 responses were recorded 3 hours after training with a 2-min cold shock given at 2-hour postconditioning. (B) Quantification of the enhanced GCaMP6 responses to the training odor (CS+) relative to the non-training odor (CS-) in the α'-lobe in OCT-trained (left panel) or MCH-trained (right panel) flies. The recordings were performed in the α'-lobe tips. The Log ratios of the CS+ response to the CS- response were calculated using the peak response amplitudes. Each value represents the mean ± SEM (n = 6 for each bar). *, p < 0.05; one-way analysis of variance followed by Tukey’s test. Genotype: +/UAS-GCaMP6m; VT30604-GAL4/+. (TIF) [file pgen.1008153.s007.tif]
